# Cross-species analysis of SHH medulloblastoma models reveals significant inhibitory effects of trametinib on tumor progression

**DOI:** 10.1038/s41420-023-01646-0

**Published:** 2023-09-19

**Authors:** Stephanie Borlase, Alexandria DeCarlo, Ludivine Coudière-Morrison, Lisa Liang, Christopher J. Porter, Vijay Ramaswamy, Tamra E. Werbowetski-Ogilvie

**Affiliations:** 1 Department of Biochemistry and Medical Genetics, Rady Faculty of Health Sciences, University of Manitoba, Winnipeg, MB Canada; 2 Department of Medical Biophysics, University of Toronto, Toronto, ON Canada; 3 The Arthur and Sonia Labatt Brain Tumour Research Centre, The Hospital for Sick Children, Toronto, ON Canada; 4 Developmental and Stem Cell Biology Program, The Hospital for Sick Children, Toronto, ON Canada; 5 Ottawa Bioinformatics Core Facility, Ottawa Hospital Research Institute, Ottawa, ON Canada; 6 Division of Haematology/Oncology, The Hospital for Sick Children, Toronto, ON Canada; 7 Texas Children’s Hospital, Houston, TX USA; 8 Department of Pediatrics, Section of Hematology-Oncology, Baylor College of Medicine, Houston, TX USA

**Keywords:** Paediatric cancer, CNS cancer

## Abstract

Sonic Hedgehog (SHH) medulloblastomas (MBs) exhibit an intermediate prognosis and extensive intertumoral heterogeneity. While SHH pathway antagonists are effective in post-pubertal patients, younger patients exhibit significant side effects, and tumors that harbor mutations in downstream SHH pathway genes will be drug resistant. Thus, novel targeted therapies are needed. Here, we performed preclinical testing of the potent MEK inhibitor (MEKi) trametinib on tumor properties across 2 human and 3 mouse SHH MB models in vitro and in 3 orthotopic MB xenograft models in vivo. Trametinib significantly reduces tumorsphere size, stem/progenitor cell proliferation, viability, and migration. RNA-sequencing on human and mouse trametinib treated cells corroborated these findings with decreased expression of cell cycle, stem cell pathways and SHH-pathway related genes concomitant with increases in genes associated with cell death and ciliopathies. Importantly, trametinib also decreases tumor growth and increases survival in vivo. Cell cycle related E2F target gene sets are significantly enriched for genes that are commonly downregulated in both trametinib treated tumorspheres and primary xenografts. However, IL6/JAK STAT3 and TNFα/NFκB signaling gene sets are specifically upregulated following trametinib treatment in vivo indicative of compensatory molecular changes following long-term MEK inhibition. Our study reveals a novel role for trametinib in effectively attenuating SHH MB tumor progression and warrants further investigation of this potent MEK1/2 inhibitor either alone or in combination with other targeted therapies for the treatment of SHH MB exhibiting elevated MAPK pathway activity.

## Introduction

Brain tumors are among the most prevalent forms of childhood cancer and account for nearly 20% of all pediatric cancer diagnoses. Medulloblastoma (MB) is the most common malignant primary pediatric brain tumor and is highly heterogeneous [1]. The current treatment consists of aggressive surgery, high doses of cytotoxic chemotherapy and radiation to the whole brain and spinal cord. Despite improved clinical outcomes, 40% of patients succumb to their disease while survivors are left with extensive cognitive and physical delays following surgery and treatment [2]. Over a decade of extensive multi-omic analyses has led to the current consensus that MB consists of at least 4 distinct molecular subgroups: WNT, Sonic Hedgehog (SHH), Group 3 and Group 4 [3–5]. These subgroups exhibit different genomic alterations, gene expression profiles, developmental cell of origin and response to treatment with additional substructure apparent within each molecular subgroup [1, 3–5]. Molecular subgrouping has improved risk stratification and provided novel opportunities to intensify therapy for the most high-risk patients and reduce therapy for those children in the lower-risk groups [6].

SHH MB is characterized by activation of the SHH signaling pathway and is comprised of very high-risk groups of both children and infants exhibiting significant intertumoral heterogeneity that often results in treatment failure despite aggressive therapy [7–13]. Recent studies have provided further insight into SHH MB substructure by defining 4 major subtypes [7, 9, 14] currently designated SHH-1, SHH-2, SHH-3, and SHH-4 [14]. Specifically, SHH-1 and SHH-2 correspond to infant subtypes while SHH-3 and SHH-4 correspond to childhood and adult subtypes, respectively. These subtypes can be further distinguished based on their driver genes and cytogenetic alterations. For example, SHH-3 tumors are enriched for *GLI2* and *MYCN* amplifications as well as *TP53* mutations which have been shown to confer a poor prognosis compared to SHH-4 tumors which often exhibit *TERT* promoter mutations, occur in older adolescents/adults, and display more favorable outcomes [9]. Targeted therapies for SHH MB are lacking, as SHH pathway inhibitors have had limited success since they are not predicted to work in young patients with tumors exhibiting mutations in downstream SHH pathway genes [15–18]. For these reasons, novel therapies that have the potential to reduce toxicity and improve survival are urgently needed.

We previously identified a role for the MAPK signaling pathway in contributing to SHH MB growth and tumor progression [19, 20]. Bioinformatics analyses of large patient datasets and tumorspheres from SHH MB cultures demonstrated that SHH MB cells exhibit elevated MAPK activity. Several highly selective and potent MEK inhibitors (MEKi) are available and are currently being tested for their therapeutic potential. For example, the MEKi selumetinib is blood brain barrier penetrant and has undergone extensive clinical testing for the treatment of other pediatric cancers such as plexiform neurofibromas [21, 22] and refractory low-grade gliomas [23, 24]. Importantly, it has shown excellent tolerability in both diseases. Selumetinib is an allosteric inhibitor that effectively inhibits enzymatic activity of MEK1/2 but does not alter the phosphorylation of MEK1/2 by Raf [25]. We previously evaluated the effect of selumetinib on SHH MB tumorigenic properties both in vitro and in vivo [19]. Selumetinib treatment decreased cell proliferation and viability in vitro at micromolar concentrations while also significantly extending survival in a preclinical xenograft model [19].

The more potent allosteric MEKi trametinib is approved for adult BRAF V600E mutant cancers and is also currently undergoing evaluation in both refractory low-grade gliomas and plexiform neurofibromas (NCT03363217, NCT02124772) [26, 27]. As opposed to selumetinib, trametinib induces MEK1/2 dissociation from C-Raf [25]. Therefore, trametinib not only inhibits MEK1/2 activity but also Raf activation of MEK1/2. Importantly, the results from recent clinical trials [28, 29] are promising and support the use of this MEKi as a potentially effective treatment for recurrent/progressive pediatric low-grade glioma, another childhood brain tumor. Thus, a preclinical evaluation of trametinib in SHH MB models is also warranted.

Here, we compared the effect of the MEKi selumetinib to trametinib on human and mouse SHH MB tumor cell properties in vitro and assessed the effect of trametinib in 3 orthotopic MB xenograft models in vivo. Trametinib significantly reduces tumorsphere size, stem/progenitor cell proliferation, viability, and cell migration in vitro at nanomolar concentrations. Trametinib also decreases tumor growth and increases survival in vivo. These data are supported by RNA-sequencing on both human and mouse trametinib treated SHH MB cells and trametinib treated xenografts that reveals significant changes in pathways associated with cell cycle, DNA repair, cell death, extracellular matrix remodeling, ciliopathies and differentiation. Our findings demonstrate a novel role for this potent MEKi in attenuating MB tumor progression and warrant further investigation into the use of trametinib alone or in combination with other targeted therapies for the treatment of SHH MB.

## Results

### Trametinib significantly inhibits SHH MB tumor properties

**Fig. 1:**
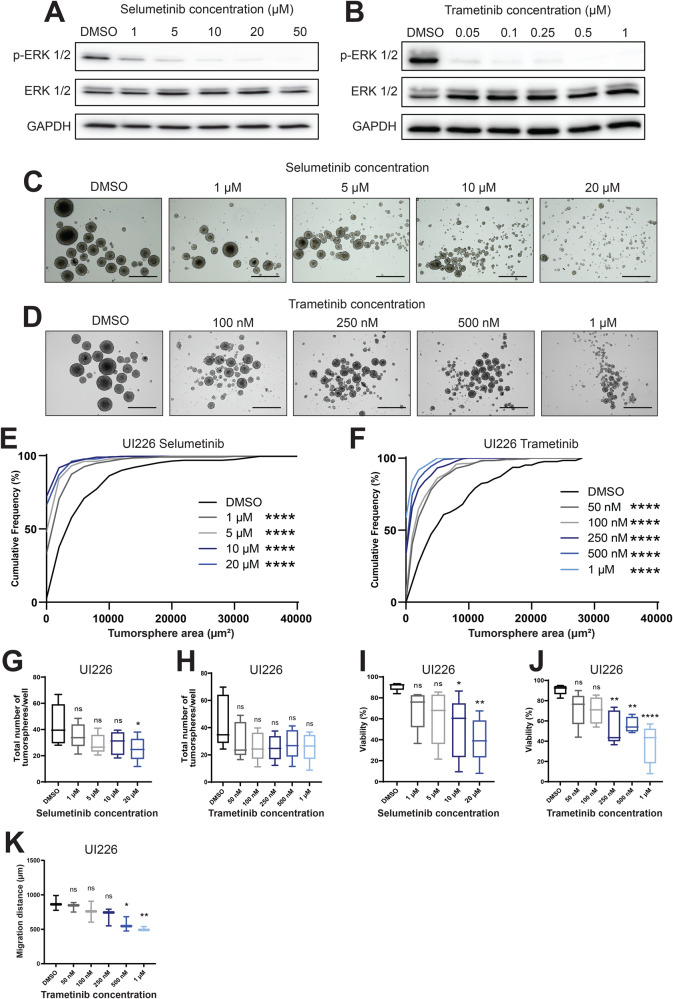
Treatment with the MEKi selumetinib or trametinib significantly impedes UI226 SHH MB tumorigenic properties in vitro. **A**, **B** Western blot for p-ERK1/2, total ERK1/2 and GAPDH following treatment of UI226 SHH MB tumorspheres with selumetinib (**A**) or trametinib (**B**) for 3 days. Total ERK 1/2 and GAPDH served as loading controls. **C**, **D** Representative images of UI226 tumorspheres following treatment with various concentrations of selumetinib (**C**) or trametinib (**D**). Scale bar: 400 µm. **E**, **F** UI226 tumorsphere size following treatment with increasing doses of selumetinib (**E**) or trametinib (**F**). Error bars: SEM. *N* = 5 biological replicates and *n* = 4 technical replicates for each biological replicate. Statistical analysis was completed using the Kolmogorov–Smirnov test. ****, *p* < 0.0001. **G**, **H** UI226 tumorsphere number following treatment with increasing doses of selumetinib (**G**) or trametinib (**H**). Error bars: SEM. *N* = 5 biological replicates and *n* = 4 technical replicates for each biological replicate. Statistical analysis was completed using the Dunnett’s test for multiple comparisons. *, *p* < 0.05. For g: DMSO vs. 20 μM, *p* = 0.0291. **I**, **J** UI226 cell viability following treatment with increasing doses of selumetinib (**I**) or trametinib (**J**). Error bars: SEM. *N* = 5 biological replicates and *n* = 4 technical replicates for each biological replicate. Statistical analysis was completed using the Dunnett’s test for multiple comparisons. *, *p* < 0.05; **, *p* < 0.01; ***, *p* < 0.001; ****, *p* < 0.0001. For **I**: DMSO vs. 10 μM, *p* = 0.0198; DMSO vs. 20 μM, *p* = 0.0025. For **J**: DMSO vs. 250 nM, *p* = 0.0012; DMSO vs. 500 nM, *p* = 0.0030; DMSO vs. 1 μM, *p* < 0.0001. **K** UI226 migration following treatment with increasing doses of trametinib. Error bars: SEM. *N* = 3 biological replicates and *n* = 4 technical replicates for each biological replicate. Statistical analysis was completed using the Dunnett’s test for multiple comparisons. *, *p* < 0.05; **, *p* < 0.01. DMSO vs. 500 nM, *p* = 0.0161; DMSO vs. 1 μM, *p* = 0.0047.

To assess the effect of trametinib on SHH MB cells, various drug concentrations were evaluated using the well-established tumorsphere assay and compared to a range of previously tested selumetinib concentrations [19]. Tumorspheres are a highly biologically relevant in vitro model system for investigating drug responses [19, 20, 30]. These 3D brain tumor cultures are grown in stem cell-enriched conditions and better sustain the genotypic and phenotypic changes as well as the transcriptional programs observed in primary tumors than adherent cells grown in serum [31, 32]. On-target effects for both selumetinib and trametinib were determined by immunoblot analyses following 3 days of drug treatment. Both selumetinib and trametinib elicit a dose-dependent decrease in p-ERK levels in UI226 tumorspheres (recently derived from a primary MB tumor [20, 33] and assigned to the SHH MB subgroup based on a 22-signature gene nanoString CodeSet developed and tested by Northcott et al., 2012 [34]) (Fig. 1A, B). In addition, both inhibitors induce the same response in Daoy [35] MB tumorspheres (Supplementary Fig. 1A, B). This decrease is observed at the lowest concentration of trametinib (50 nM) in tumorspheres from both SHH MB cell lines.

Next, the effects of selumetinib and trametinib on tumorsphere size were independently evaluated by cumulative frequency distribution analyses using Fiji/ImageJ. Both MEKi significantly decrease UI226 and Daoy tumorsphere size (Fig. 1C–F, Supplementary Fig. 1C–F). A significant effect on UI226 tumorsphere number is observed at the highest concentration of selumetinib; however, only Daoy primary tumorsphere number is significantly decreased with trametinib treatment at 250 nM and above (Fig. 1G, H, Supplementary Fig. 1G, H). Both inhibitors significantly decrease UI226 and Daoy cell viability (Fig. 1I,J, Supplementary Fig. 1I, J) with notable decreases starting at 10 μM for selumetinib and 250 nM for trametinib.

Having shown a strong inhibitory effect of trametinib on tumorspheres in the nanomolar range, we next assessed the effect of this more potent MEKi on cell migration in our 3D collagen assay. Both UI226 and Daoy exhibit a significant decrease in cell motility following treatment with trametinib (Fig. 1K, Supplementary Fig. 1K).

**Fig. 2:**
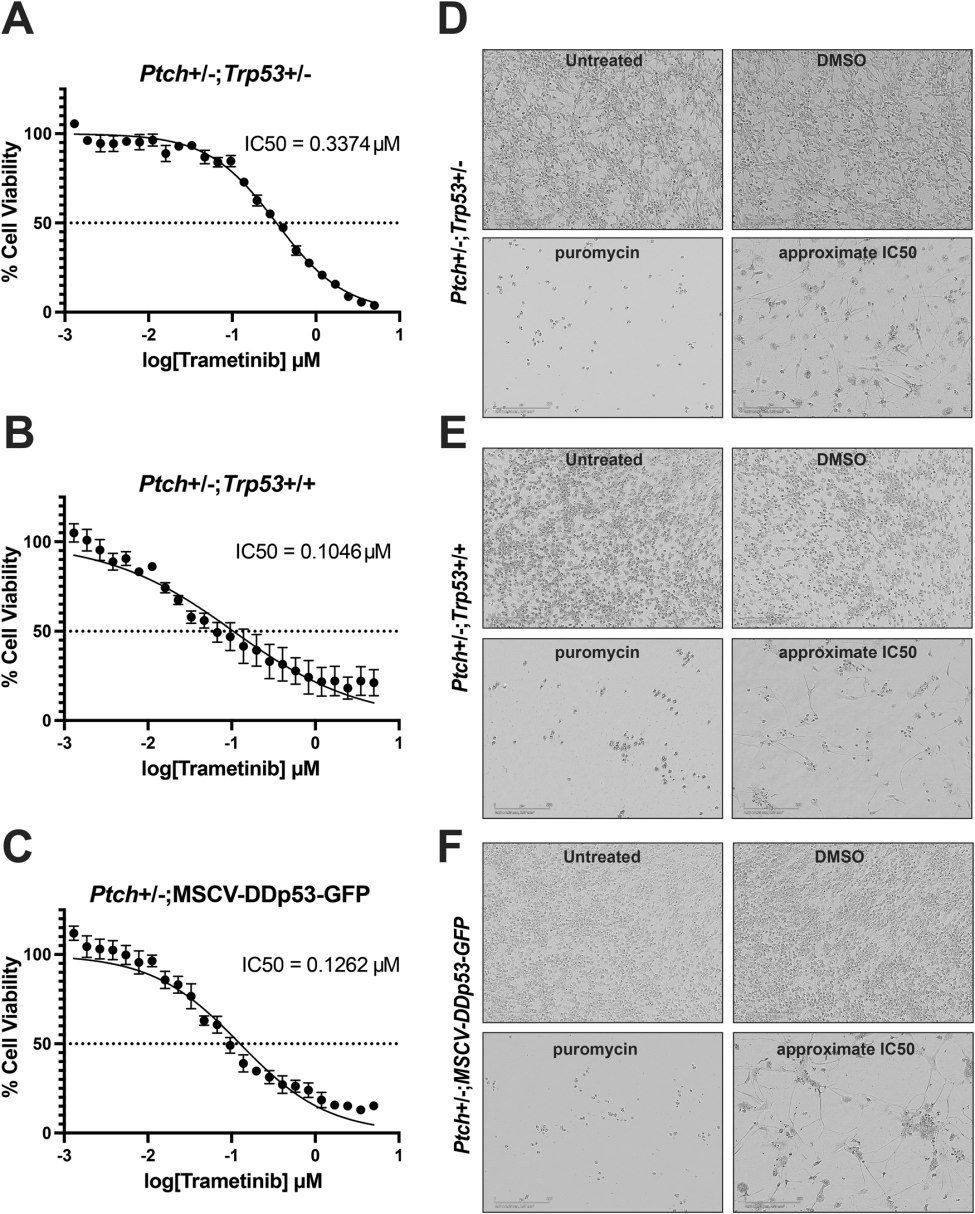
Trametinib decreases SHH MB mouse cell viability. **A**–**C** Dose response curves for SHH MB mouse cells obtained from *Ptch*±:*p53*± (**A**), *Ptch*±:*p53* + /+ (**B**), and *Ptch*±; MSCV-DDp53-GFP (**C**) tumors. Data for each cell model represent 2 biological replicates completed in duplicate. Error bars: SEM. **D**–**F** Representative images of SHH MB mouse cells obtained from *Ptch*±:*p53*± (**D**), *Ptch* ±:*p53* + /+ (**E**), and *Ptch*±;MSCV-DDp53-GFP (**F**) tumors that are untreated or treated with DMSO (negative control), puromycin (positive control for cell death) or trametinib. Images shown for trametinib represent the phenotype at the approximate IC50 for each set of cells. Scale bar: 200 μm.

To complement our findings in human SHH MB cells, we used a cross-species approach and tested trametinib on a series of recently derived low passage primary cultures from genetically engineered mouse SHH MB models including cells obtained from fresh *Ptch*±:*p53*±, *Ptch*±:*p53* + /+, and *Ptch*±; MSCV-DDp53-GFP tumors. These mouse SHH MB cells can be expanded in culture and retain SHH signaling in modified stem-cell enriched conditions [36, 37]. Indeed, significant reductions in viability are observed following 96 h trametinib treatment with IC50s in the 105–337 nM range. (Fig. 2A–C). In addition to changes in viability, all 3 SHH MB cell cultures exhibit phenotypic changes indicative of differentiation such as a flattened morphology and increases in cell extensions (Fig. 2D–F). Collectively, these results demonstrate that trametinib is highly effective at inducing an overall decrease in both human and mouse SHH MB tumorigenic properties at lower concentrations in vitro.

### RNA sequencing reveals significant changes in cell cycle and differentiation pathways following MEK inhibition

**Fig. 3:**
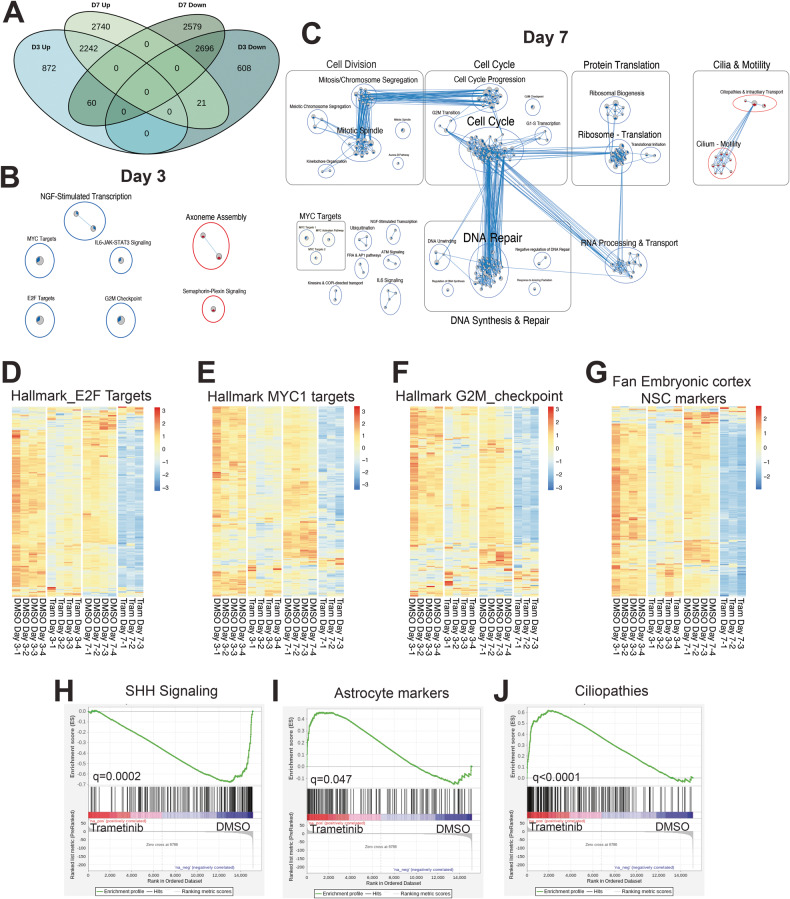
RNA sequencing reveals significant changes in cell cycle and differentiation pathways following MEK inhibition. **A** Venn diagram depicting the number of significantly differentially expressed genes that are unique to one timepoint (day 3 or day 7) or that overlap between both timepoints following trametinib treatment. **B**, **C** Enriched pathways and biological processes as determined by GSEA following 3 days (**B**) or 7 days (**C**) trametinib treatment of UI226 tumorspheres. GSEA results for Hallmark, Canonical Pathways (C2:CP), and Gene Ontology (C5) (*q* value < 0.01) were used to build the enrichment map. Node coloring is based on the normalized enrichment score (NES), with blue representing enrichment in vehicle and red representing enrichment in trametinib samples. **D**–**G** Heat maps depicting genes associated with E2F targets (**D**), MYC targets (**E**), G2M checkpoint (**F**), as well as neural stem cell markers (**G**) that are enriched in downregulated genes following trametinib treatment (negative NES in GSEA analysis). Significant enrichment was observed at day 3, and this was further enhanced at day 7. For Hallmark G2M_checkpoint Day 3, *q* = 0.0087. For all other categories and timepoints, *q* < 0.0001. **H**–**J** GSEA results for gene sets upregulated in GCNPs after SHH stimulation (**H**), markers for embryonal cerebral cortex astrocytes, (**I**) or ciliopathy markers (**J**) that are enriched in downregulated (**H**) or upregulated (**I**, **J**) genes following 7 days of trametinib treatment. padj < 0.05* for all signatures. For GCNP after SHH stimulation in (**H**): Day 7, *q* = 0.0002, NES = −1.84. For astrocyte signature in (**I**): Day 7, q = 0.047, NES = 1.62. For cilopathy signature in (**J**): Day 7, *q* < 0.0001, NES = 2.26.

To interrogate the molecular mechanisms associated with the decrease in tumorigenic properties following trametinib treatment, we performed RNA sequencing (RNA-seq) on UI226 SHH MB tumorspheres to characterize early (3 days) relative to the late or sustained (7 days) transcriptomic changes following trametinib treatment in vitro. Initial assessments of cell death in our tumorsphere assay revealed a significant decline in survival at 250 nM trametinib treatment. Therefore, this concentration was chosen for RNA-seq analyses. Overall gene expression patterns are consistently different in the trametinib-treated samples relative to the DMSO controls (Supplementary Fig. 2A, B). Comparison of day 3 and day 7 treated tumorspheres reveals significant overlap between timepoints (Fig. 3A). Of the 6499 significantly differentially expressed genes (padj <0.05) following 3 days trametinib treatment, 76% (4938 genes) were similarly up (2242 genes) or downregulated (2696 genes) at day 7 (Fig. 3A). Hallmark gene sets associated with E2F and MYC targets as well as G2M checkpoint and neural stem cells are among the most significantly enriched downregulated gene sets following trametinib treatment at both timepoints (Fig. 3C–G, Supplementary Fig. 3A–H, Supplementary Data 1). These results provide support for our in vitro assays demonstrating growth-related inhibitory effects on our stem cell enriched tumorsphere populations. The differences observed at day 3 are further enhanced at day 7 (Fig. 3D–G). In addition, genes associated with SHH-stimulated granule neuron progenitor cell proliferation are enriched in gene sets that are downregulated following 7 days of trametinib treatment (Fig. 3H, Supplementary Fig. 3I, Supplementary Data 2). In contrast, genes associated with astrocytic differentiation and ciliopathies are significantly enriched in gene sets that are upregulated specifically at day 7 (Fig. 3I–J, Supplementary Fig. 3J-K, Supplementary Data 2) suggesting an overall shift in cell fate decisions.

**Fig. 4:**
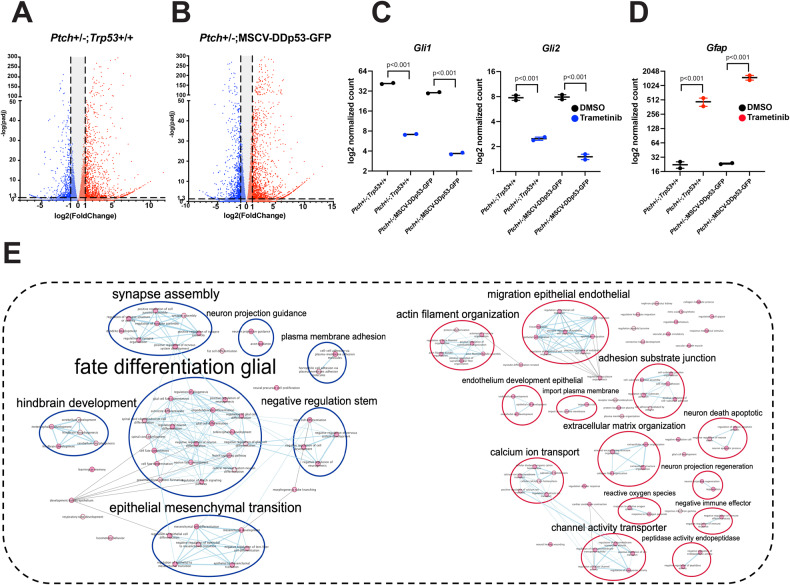
Trametinib treatment alters pathways associated with cell death, differentiation, and extracellular matrix remodeling in isogenic mouse SHH MB cell models. **A**, **B** Volcano plots depicting the log2 fold change and *p* values in RNA-seq data comparing trametinib (*N* = 2) to vehicle controls (*N* = 2) in *Ptch* + /−:*p53* + /+ (**A**) and *Ptch* + /−;MSCV-DDp53-GFP (**B**) cells (FDR < 0.05). **C**, **D** Normalized counts depicting changes in *Gli1* and *Gli2* (**C**) or *Gfap* (**D**) expression following trametinib treatment. Error bars: SEM. *p* < 0.001*** for all genes. Blue represents downregulated and red represents upregulated genes. **E** Pathway enrichment of RNA-seq data 96 h after treatment with 200 nM trametinib. Red represents upregulated pathways and blue represents downregulated pathways. Combined analysis demonstrates 1476 common differentially expressed genes (DEGs) between *Ptch* + /−:*p53* + /+ and *Ptch* + /−;MSCV-DDp53-GFP cells with 1069 being upregulated and 407 being downregulated. Nodes represent pathways and edges connect pathways with overlapping genes. Nodes are connected by an edge if they share gene set similarity ≥ 0.375. Nodes are manually laid out to form a clearer picture. Clusters of related nodes are given a general pathway label using the AutoAnnotate plug-in (v.1.3).

To complement these findings, we also performed RNA-seq on the isogenic *Ptch* + /−:*p53* + /+, and *Ptch* + /−; MSCV-DDp53-GFP tumor cells treated with 200 nM trametinib or vehicle for 5 days (Supplementary Data 3). We observed similar transcriptome changes for *Ptch* +/-:*p53* + /+ and *Ptch* + /−; MSCV-DDp53-GFP tumor cells. (Fig. 4A, B). The downstream SHH pathway genes *Gli1* and *Gli2* were significantly downregulated while the astrocytic marker glial fibrillary acidic protein (*Gfap*) was significantly upregulated (Fig. 4C, D). Moreover, g:profiler analyses revealed significant enrichment of extracellular matrix (ECM)-related and actin filament organization as well as neuron apoptotic death in upregulated gene sets following trametinib treatment (Fig. 4E). In contrast, gene sets associated with glial fate differentiation and epithelial to mesenchymal transition were downregulated following trametinib treatment (Fig. 4E). Collectively, the transcriptomic changes following trametinib treatment demonstrate significant and sustained alterations in cell cycle, cell death and differentiation-related pathways, as well as genes associated with SHH pathway signaling in human and mouse SHH MB tumor cells.

### MEK1/2 inhibition significantly increases survival and reduces tumor growth in MB xenograft models

**Fig. 5:**
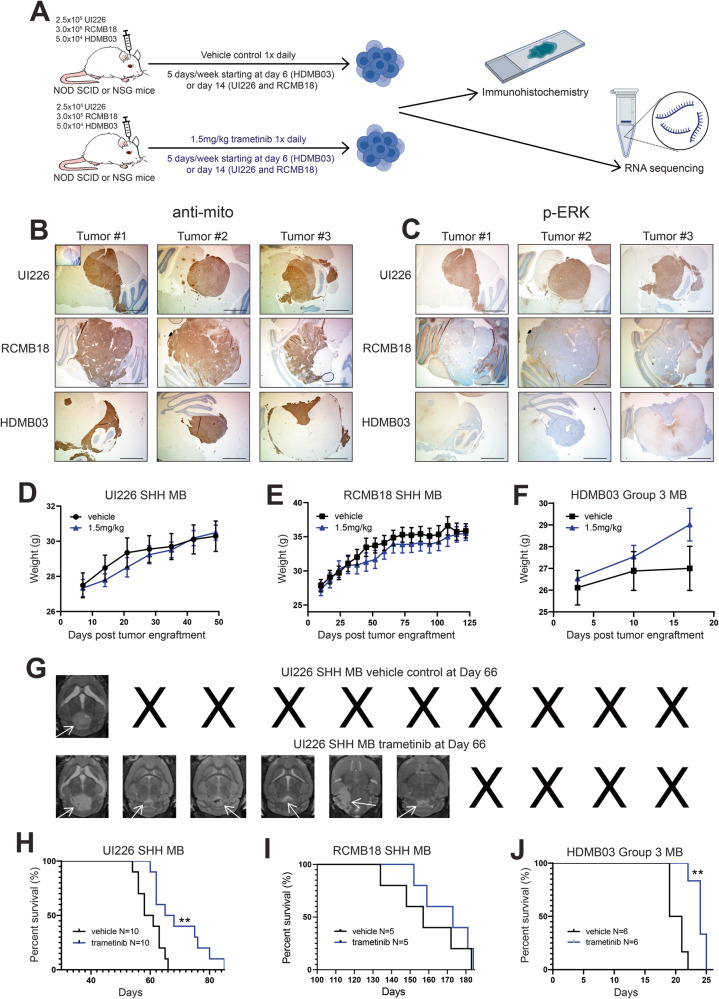
MEK1/2 inhibition significantly increases survival and reduces tumor growth in MB xenograft models. **A** Schematic outlining the 3 xenograft mouse model experiments. 2.5 × 10^5^ cells/animal (UI226), 3 × 10^5^ cells/animal (RCMB18) or 5 × 10^4^ cells/animal (HDMB03) were injected into the cerebellum of immunodeficient NOD/SCID or NSG mice. Trametinib was administered via oral gavage on a 5 day on, 2 day off schedule following tumor engraftment. Treatment started 6 days after tumor engraftment for the HDMB03 cells and 14 days after tumor engraftment for the UI226 and RCMB18 cells. **B**, **C** Representative images of immunohistochemical staining for anti-mitochondria antibody (**B**) and p-ERK antibody (**C**) in FFPE tissue sections derived from 3 representative independent control tumors from UI226 SHH MB, RCMB18 SHH MB and HDMB03 Group 3 MB xenografts. Scale bar: 1550 μm. **D**–**F** NOD SCID and NSG mouse weights over time following vehicle control (black) or 1.5 mg/kg trametinib treatment (blue) post tumor engraftment with UI226 SHH MB cells (*N* = 10 mice for each treatment group) (**D**), RCMB18 SHH MB cells (*N* = 5 mice for each treatment group) (**E**) or HDMB03 Group 3 MB cells (*N* = 6 mice for each treatment group) (**F**). Drug treatment was initiated at day 14 following injection of UI226 and RCMB18 tumor cells and at day 6 following injection of HDMB03 tumor cells. Error bars: SEM. **G** Representative MRI images of UI226 SHH MB tumors in NOD SCID mice treated with vehicle control or 1.5 mg/kg trametinib at day 66 post-surgery. **H**–**J** Kaplan Meier curves following transplantation of NOD SCID mice with 2.5 × 10^5^ UI226 SHH MB cells (*N* = 10 mice for each treatment group) (**H**), NSG mice with 3.0 × 10^5^ RCMB18 SHH MB cells (*N* = 5 mice for each treatment group) (**I**), and NOD SCID mice with 5.0 × 10^4^ HDMB03 Group 3 MB cells (*N* = 6 for each treatment group) (**J**). Mice were treated with vehicle control or 1.5 mg/kg of trametinib. *P* value was determined using the log-rank test. **, *p* < 0.01. For UI226 in (**H**): *p* = 0.0077. For HDMB03 in (**J**): *p* = 0.0012. Treatment started 14 days following UI226 and RCMB18 tumor cell injection and 6 days following HDMB03 tumor cell injection. Animals were treated once daily, 5 days a week by oral gavage, with a 2 day drug holiday on weekends until they reached endpoint.

Trametinib significantly decreases tumorigenic properties of SHH MB cells in vitro and is also known to be blood brain barrier penetrant [26]. We therefore tested the effect of this potent MEKi in xenograft mouse models (Fig. 5A). First, immunohistochemistry (IHC) was performed on formalin-fixed paraffin-embedded (FFPE) tissue sections derived from control tumors from 3 MB xenograft models to determine potential efficacy in vivo. This includes our UI226 SHH MB model and the recently derived RCMB18 SHH MB PDX model that exhibits loss of *TP53*, *MYCN* amplification and a *SMO* mutation [18, 38]. We also evaluated HDMB03 Group 3 MB xenografts to determine subgroup specificity and potential responsiveness in a model of the most highly aggressive MB. Three tumors from each MB model were stained with an anti-mitochondria antibody to specifically visualize human cells in NOD SCID mice (Fig. 5B). Sequential sections from each xenograft model were then stained for p-ERK (Fig. 5C, Supplementary Fig. 4A). Interestingly, UI226 SHH MB tumor cells are all strongly positive for p-ERK compared to RCMB18 SHH MB tumors which exhibit much lower positive p-ERK staining (Fig. 5C, Supplementary Fig. 4A). Group 3 HDMB03 MB xenografts also exhibit lower levels of p-ERK (Fig. 5C, Supplementary Fig. 4A). These results suggest that the UI226 SHH MB xenograft model would be the most responsive to trametinib treatment in vivo.

Next, a pilot study was completed to evaluate trametinib toxicity in NOD SCID mice using 0.5 mg/kg, 1.0 mg/kg, and 1.5 mg/kg, based on concentrations previously chosen in other brain tumor models [39]. At all 3 concentrations, the mice show no signs of toxicity and consistently gain weight over 28 days of treatment (Supplementary Fig. 4B). Thus, we chose 1.5 mg/kg of trametinib for further studies in all 3 xenograft models.

UI226 SHH MB (2.5 × 10^5^ cells/animal), RCMB18 SHH MB PDX cells (3 × 10^5^ cells/animal) and Group 3 MB HDMB03 (5 × 10^4^ cells/animal) tumorspheres were dissociated and injected into the cerebellums of immunodeficient NOD SCID or NSG mice. Trametinib was then administered, via oral gavage, on a 5-day on, 2-day off schedule until endpoint was reached. This drug holiday on weekends is associated with highly favorable toxicity profiles [40], and as anticipated, weights steadily increased overtime following trametinib treatment (Fig. 5D–F). MRI analysis of UI226 SHH MB tumors 66 days post-tumor cell injection revealed that the 6 remaining trametinib treated mice harbor tumors of varying size with 4 animals still exhibiting small tumors compared to the lone vehicle control (Fig. 5G). This translated to an overall significant increase in survival of UI226 SHH MB trametinib treated xenografts (Fig. 5H). RCMB18 SHH MB xenografts did not exhibit a significant increase in survival (Fig. 5I) which is likely attributed to their lower levels of MAPK pathway activity (Fig. 5C). However, a trend towards increased survival is observed (median survival of 173 days in the trametinib group relative to 157 days in the vehicle controls). HDMB03 MB xenografts also display similarly low levels of p-ERK; however, trametinib treatment resulted in a small but statistically significant survival increase in this highly aggressive Group 3 model (Fig. 5J). Collectively, these results support our in vitro studies and corroborate our IHC findings demonstrating robust MAPK activity particularly in the UI226 SHH MB xenografts. These results demonstrate the potential clinical utility of trametinib for treating MB tumors with elevated MAPK pathway activity.

### Pathways associated with cell cycle, DNA repair and survival are differentially expressed following trametinib treatment in vivo

**Fig. 6:**
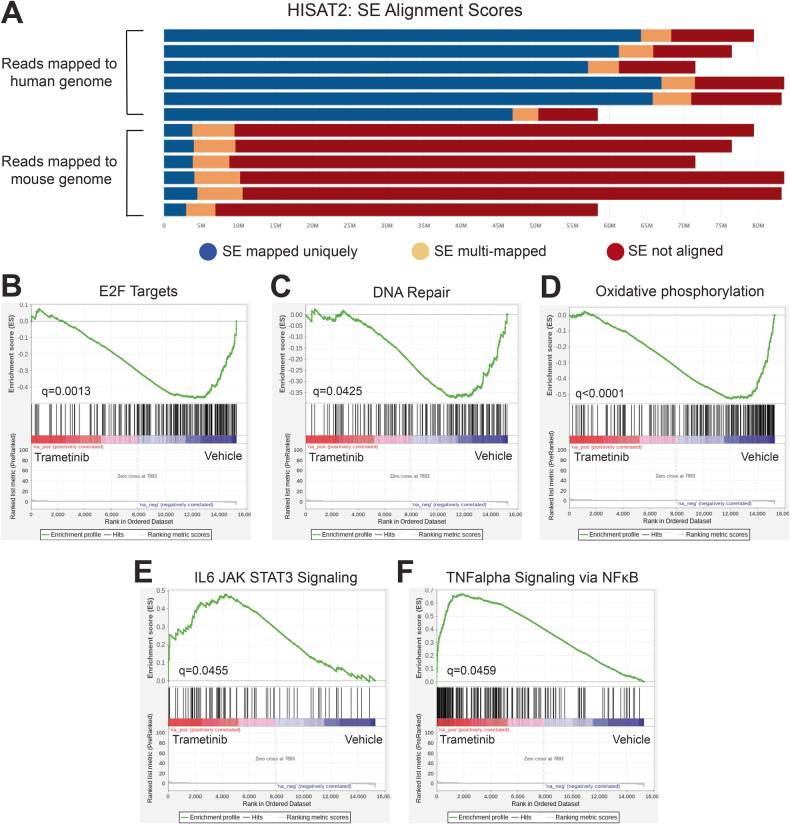
Molecular pathways are differentially expressed following trametinib treatment in vivo. **A** Reads mapping to human and mouse genomes. XenofilteR was performed to remove the mouse reads. **B**–**D** GSEA results for gene sets comprising E2F targets (**B**), DNA repair (**C**) and oxidative phosphorylation (**D**) showing enrichment for genes that are downregulated following trametinib treatment. padj < 0.05* for all signatures. For E2F targets, *q* = 0.0013, NES = −1.92. For DNA repair, *q* = 0.0425, NES = −1.49. For oxidative phosphorylation, *q* < 0.0001, NES = −2.18. **E**, **F** GSEA results for gene sets comprising IL6/JAK/STAT3 signalling (**E**) and TNFalpha signaling (**F**) showing enrichment for genes that are upregulated following trametinib treatment. padj < 0.05* for all signatures. For IL6/JAK/STAT3 signaling, *q* = 0.0455, NES = 1.53. For TNFalpha signaling via NFκB, *q* = 0.0459, NES = 2.43.

Next, RNA-seq was performed to interrogate the transcriptome changes following trametinib treatment in our responsive UI226 MB xenograft model. Endpoint tumors from UI226 SHH MB xenografts were extracted from trametinib treated mice (*N* = 3) and vehicle controls (*N* = 3), and RNA-seq libraries were constructed and sequenced. XenofilteR was applied to remove reads that mapped to the mouse genome (Fig. 6A) prior to analysis of expression differences. The vehicle and trametinib treated tumors are similar at endpoint, as only 113 genes are significantly (*p* < 0.05) differentially expressed (Supplementary Data 4). However, in line with our human tumorsphere data, GSEA reveals that the hallmark E2F target gene set has a significant negative normalized enrichment score (NES = −1.92), indicating that these genes are predominantly expressed at a lower level in trametinib treated xenografts (Fig. 6B). Interestingly, the DNA Repair (NES = −1.49) and oxidative phosphorylation (NES = −2.18) hallmark gene sets are also significant for genes that are downregulated following trametinib treatment (Fig. 6C, D). Similar to our previous findings following selumetinib treatment in vivo [40], the hallmark JAK/STAT3 and TNFα/NFκB signaling pathway gene sets each have a significant positive NES (1.53 and 2.43, respectively) and are thus enriched for genes that are upregulated in trametinib treated xenografts (Fig. 6E, F). These results demonstrate that trametinib inhibits cell cycle, DNA repair, and metabolic pathways following long-term MEKi treatment. However, this is also accompanied by upregulation of cell signaling pathways that may be compensating for MEK inhibition in vivo.

## Discussion

Targeting MB tumor cells through MEK inhibition represents a novel and rational therapeutic strategy for the treatment of SHH MB. Trametinib is currently in clinical trials for the treatment of refractory low-grade gliomas and plexiform neurofibromas and has been approved for adult BRAF V600E mutant cancers [26, 27]. Our cross-species approach using multiple human and mouse SHH MB tumor cell models demonstrates that this more potent MEKi significantly abrogates MB tumor progression. Trametinib inhibits growth, viability, and migration in vitro while also increasing survival in 2 different MB xenograft mouse models using much lower concentrations than what was previously employed for selumetinib. Together, these results warrant further investigation of trametinib as a novel therapeutic strategy for MB either as a single agent or in combination with other targeted therapies.

We recently identified molecular pathways that could be compensating for selumetinib treatment, thus contributing to molecular adaptation and drug resistance in vivo [40]. JAK/STAT3 pathway activation is upregulated in SHH MB tumor cells following treatment with selumetinib. Importantly, we showed that combined treatment with selumetinib and pacritinib, a JAK/STAT3 pathway inhibitor, effectively decreases SHH MB tumor progression. As we also observed upregulation of genes associated with JAK/STAT3 signaling in trametinib treated xenografts, this may represent a conserved mechanism of molecular adaptation following long-term MEKi in our models. Future studies will evaluate the effect of JAK/STAT3 pathway inhibition in combination with trametinib on tumorigenic properties both in vitro and in vivo. In addition, TNFα/NFκB gene sets were enriched for genes that are upregulated in trametinib treated xenografts. Therefore, additional studies will also evaluate the effect of trametinib in combination with a TNFα/NFκB pathway inhibitor. Finally, the combination of trametinib with other standard of care chemotherapeutic agents will also be explored.

Immunohistochemistry staining of tissue sections derived from different MB xenografts revealed variable p-ERK levels and therefore MAPK signaling activity across models. UI226 SHH MB tumors exhibited universally high p-ERK levels compared to the RCMB18 SHH MB and HDMB03 Group 3 tumors. The in vivo data supported these findings, as the most significant increase in survival was observed in the UI226 SHH MB xenografts following trametinib treatment. While there was also a modest improvement in RCMB18 SHH MB survival, the increase was not significant. This could be attributed to molecular adaptation over the very long course of tumor progression in this model combined with the overall lower MAPK pathway activity. However, despite low p-ERK levels, we did observe a small, but significant increase in survival following MEKi treatment in the HDMB03 *MYC*-amplified Group 3 MB model. As hallmark gene sets associated with MYC targets were significantly downregulated following trametinib treatment in our RNA-seq data, MEKi may converge on critical MYC-targets in Group 3 MBs as well. Alternatively, our previous spatial profiling studies have shown evidence of suppression through pathway crosstalk as significant decreases in proteins associated with PI3K/Akt signaling following selumetinib treatment were also observed [40]. Signaling crosstalk may be a contributing factor to the survival difference observed in the *MYC*-amplified Group 3 MB xenografts.

An interesting finding in our RNA-seq tumorsphere data was the dysregulation of SHH signaling and enrichment of genes associated with ciliopathies or primary cilia following trametinib treatment. Primary cilia are essential for vertebrate SHH signal transduction [41] and have been shown to play critical roles in cerebellar granule neuron progenitor cell (GNPC) proliferation [42–44] as well as MB tumor progression and drug resistance [42, 45, 46]. In our study, gene sets upregulated in SHH-stimulated GNPC proliferation were enriched in genes that are downregulated following 7 days of trametinib treatment. Significant decreases in the SHH pathway effector genes, *Gli1* and *Gli2*, were also observed in both mouse models. In contrast, genes associated with ciliopathies were enriched in gene sets that were upregulated following trametinib treatment. While likely reflecting the sustained, but indirect, effects of long term MEKi treatment, these results are interesting, as it is well known that SHH signaling through the primary cilium is essential for GNPC proliferation in the external granular layer of the developing cerebellum [42]. Moreover, primary cilia are either required for or inhibit SHH MB tumor formation depending on the underlying oncogenic event in the SHH pathway [45]. MEK/ERK signaling drives SHH pathway inhibitor resistance and promotes metastasis in SHH MB mouse models [37]. While loss of primary cilia is associated with drug resistance to SHH pathway inhibitors in MB [46], the specific relationship between MEK/ERK signaling and primary cilia or genes associated with primary cilia in SHH MB is not well known. Indeed, a recent study showed that smoothened inhibitor-resistant basal cell carcinomas exhibit MAPK pathway enrichment concomitant with increases in ciliome gene mutations and reductions in primary cilia [47] suggesting that the primary cilia control SHH-to-MAPK pathway switching.

Our study reveals a novel role for trametinib in effectively attenuating SHH MB tumor progression and supports the potential clinical utility of this potent MEKi to treat MB tumors with elevated MAPK pathway activity. Trametinib either alone or in combination with other upregulated signaling pathway inhibitors or chemotherapeutic agents could be translated into clinical trial development to generate new and innovative targeted therapies to treat these very aggressive pediatric brain tumors.

## Materials and methods

### Human cell culture and in vitro drug treatments

Experiments were performed using UI226 low passage primary SHH MB cells as well as the established cell line Daoy [35]. UI226 cells were a gift from Dr. Timothy Ryken (Dartmouth-Hitchcock Medical Center, New Hampshire, USA) and were propagated in StemPro media as previously described [19, 33]. NanoString analysis was used to classify UI226 cells as SHH MB based on a previously established 22-signature gene CodeSet developed and tested by Northcott et al., 2012 for MB molecular subgrouping [34]. Daoy cells were purchased from ATCC and cultured as previously described [19, 20]. STR profiling (ATCC) was used to authenticate all cell lines utilized in this study. Cell lines were not tested for mycoplasma. The tumorsphere assay was used to evaluate the effect of selumetinib and trametinib on both cell lines. Briefly, UI226 (5000 cells/well) and Daoy (10,000 cells/well) cells were plated into each well of a 24-well ultra-low attachment plate (Corning, New York, USA) and grown in StemPro media [19, 33] (UI226) or neural precursor media (Daoy) consisting of DMEM F12, B27 [50x], N2 (100×), 10 ng/mL bFGF and 10 ng/mL EGF. Selumetinib (MedChemExpress, Monmouth Junction, New Jersey, USA) was added at 1 µM, 5 µM, 10 µM, and 20 µM to cells while trametinib (MedChemExpress) was added at 50 nM, 100 nM, 250 nM, 500 nM or 1 µM to cells. DMSO served as the vehicle control. Tumorspheres were incubated undisturbed at 37 °C, 5% CO_2_ for 7 days (UI226) or 5 days (Daoy), counted and measured. Tumorsphere number, tumorsphere size, cell viability and total cell number were evaluated and compared to DMSO controls. Fiji/ImageJ was used to evaluate individual tumorsphere size in multiple wells per treatment group. Results were displayed as cumulative frequency distribution of tumorsphere area. Only tumorspheres >25 μm were included in these analyses [40].

For immunoblotting analysis, 2 × 10^5^ cells/well were plated into each well of a 6-well ultra-low attachment plate in the presence of drug as described above. Cells were collected after 3 days, and protein was extracted.

### Immunoblotting

UI226 and Daoy tumorspheres were treated with selumetinib or trametinib as described above. Protein was isolated from dissociated primary tumorspheres using Lysis Buffer (25 mM Tris pH 7.4, 150 mM NaCl, 1% Triton X-100, 5 mM EDTA), plus 1x Protease Inhibitor Complex (Roche), and 0.5 mM of sodium orthovanadate. Protein quantities ranging from 20 to 30 μg were loaded onto 12% Tris-glycine gels and resolved by SDS–PAGE. Protein was transferred using a semi-dry transfer method to nitrocellulose membranes (Bio-Rad) and washed as previously described [19, 20]. Membranes were blocked with 5% milk in 1× TBST (20 mM Tris, 137 mM NaCl, 0.5% Tween 20, pH 7.6) for 1 h at room temperature and then incubated overnight at 4 °C in primary antibodies (Supplementary Table 1). The following day, membranes were washed 3× with 1× TBST for 5 min and then secondary antibodies (Supplementary Table 1) conjugated to horseradish peroxidase were added for 1 h at room temperature. Signal detection was performed using SuperSignal West Pico (ThermoFisher Scientific, Waltham, Massachusetts, USA) and blots were imaged using a Fusion FX Vilber Lourmat chemiluminescent imaging system (Marne LA Vallee Cedex 3, France). All original immunoblots are shown in *the Original Data File*.

### Migration assay

Daoy and UI226 cells were plated in 96-well ultra-low attachment round-bottom plates at 10,000 cells/well and 4000 cells/well, respectively and left to aggregate or form spheroids over 4 days. They were then overlain with a Type I collagen mixture (Collagen type I (Corning, New York, USA), DMEM, and NaOH). Once the collagen had solidified, 100 µL of medium containing DMSO or 50 nM, 100 nM, 250 nM, 500 nM or 1 μM of trametinib was added. Migration was assessed after 3 days by subtracting the day 0 diameter from the diameter of the migration front measured at day 3. Measurements were taken using a Zeiss Primovert inverted microscope.

### Mouse SHH MB dose response curves

Three different SHH MB tumor models, including cells obtained from fresh *Ptch* + /−:*p53* + /−, *Ptch* + /−:*p53* + /+, and *Ptch* + /−;MSCV-DDp53-GFP SHH mouse tumors, were cultured and expanded as previously described [36, 40]. For generation of dose response curves, cells were plated on laminin-coated 96-well plates at 3000 cells/well in duplicate, left to adhere for 24 h and then treated with 24 different concentrations of trametinib ranging from 13 nM to 5 μM. Following 96 h of treatment, AlamarBlue was added at 10% of volume. Fluorescence was read using a plate reader and cell viability was assessed by fluorescent intensity. Data were analyzed using GraphPad Prism Software and line of best fit was calculated by a non-linear regression (dose-response log(inhibitor) vs. normalized response – variable slope). Data were normalized to percent cell viability based on the raw fluorescent intensity. Controls included four wells each of untreated, DMSO (negative) and puromycin (positive).

### Intracerebellar transplantation and drug treatment

All procedures were approved by the University of Manitoba Animal Care Committee. UI226 SHH MB cells were cultured as tumorspheres, dissociated, and 2.5 × 10^5^ cells/animal were injected into the cerebellum of 7–9 week old male immunodeficient NOD SCID mice. For all experiments, *N* = 5–10 animals were utilized for each treatment group and randomly assigned based on previously published studies as well as our experience with these models to detect statistical differences between groups [19, 20, 30, 48–50]. Group 3 MB HDMB03 cells were obtained from Dr. Till Milde [51], cultured as previously described [32] and 5 × 10^4^ cells/animal were injected into the cerebellum of 7–9 week old immunodeficient NOD SCID mice. RCMB18 cells were kindly provided by Dr. Robert Wechsler-Reya. This PDX model exhibits loss of *TP53*, *MYCN* amplification and a *SMO* mutation [38, 52] designating RCMB18 as SHH MB. Patient derived SHH MB RCMB18 cells were thawed and 3 × 10^5^ cells/animal were injected into the cerebellum of immunodeficient NOD SCID IL2Rg null (NSG) mice. Following tumor engraftment, the animals were randomly divided into two groups with one receiving either 0.5% hydroxypropyl methyl cellulose, 0.1% polysorbate 80 as the vehicle control or 1.5 mg/kg of trametinib. Treatment was administered via oral gavage on a 5 days on, 2 days off schedule following tumor engraftment. Animals were treated until they reached endpoint (mice could no longer walk or groom themselves properly or had lost 20% of their peak weight). Animals were then perfused and brains were extracted for histological analysis. The mice were held according to the Guidelines of the Canadian Council on Animal Care and the Animal Care and Use Policy of the University of Manitoba. Briefly, they were kept housed in IVC caging (Tecniplast) with bedding that had been sterilized by steam autoclave. The light cycle of the room began with lights on at 6:00 a.m. and continued on a 12 h on and 12 h off schedule. The room temperature was kept between 21 and 23 °C and had a humidity target of 50%. MRI was performed on a MR Solutions cryogen free FlexiScan 7 T system (MR Solutions, Guildford, Surrey, UK) as previously described and personnel blinded to treatment groups [19, 32]. During the procedure, mice were anaesthetized with 4% isoflurane and maintained with a mask at 1.5–2% isoflurane in oxygen.

### RNA-sequencing on human UI226 tumorspheres and xenografts

To identify molecular changes following trametinib treatment, we performed RNA-sequencing (RNA-seq) on drug treated UI226 SHH MB tumorspheres as well as on UI226 xenografts. UI226 tumorspheres were plated at 2 × 10^5^ cells/well into each well of a 6-well ultra-low attachment plate. DMSO control or 250 nM of trametinib was added to the tumorspheres and samples were collected after 3 days (*N* = 4) or 7 days (*N* = 3–4) for RNA extraction. For the in vivo study, once UI226 xenograft mice reached endpoint, tumors were resected, and human cells were isolated using a brain tumor dissociation kit (Miltenyi Biotec, Bergisch Gladbach, North Rhine-Westphalia, Germany) followed by a mouse cell depletion kit (Miltenyi Biotec) according to manufacturer’s instructions. RNA was extracted from 3 vehicle control endpoint tumors taken at days 61, 63 and 65 and from 3 trametinib treated xenografts at endpoint taken at days 75, 76 and 80. RNA was extracted using the Norgen RNA extraction kit (Norgen Biotek, Thorold, ON, Canada) according to manufacturer’s instructions. Library preparation and RNA-seq were performed by StemCore laboratories at the Ottawa Hospital Research Institute (Ottawa, ON, Canada) as previously described [19, 32]. Libraries were prepared from 500 ng of input total RNA with stranded mRNA library prep kit (Illumina). RNA-seq was performed comparing control and trametinib treated tumor cells from primary xenografts.

### Human xenograft and tumorsphere RNA-sequencing analysis

Reads from xenograft tumor RNA-seq libraries were mapped to the human reference genome (GRCh38 assembly) guided by transcripts from GENCODE release 35, and separately to the mouse reference genome (GRCm38 assembly) guided by transcripts from GENCODE release M25, using hisat2 2.2.1. Human and mouse BAM files were passed to XenofilteR (v1.6) [53] to identify and remove reads that map more closely to the mouse genome, and that are thus considered to originate from the mouse host. The remaining (presumed human graft origin) reads were quantified with salmon v.1.4.0 [54] against an index built from the GENCODE v35 assembly with inclusion of genomic decoy sequences. Data were loaded into R using the tximport library and the gene/count matrix was filtered to retain only genes with five or more mapped reads in two or more samples. Differential expression was assessed using DESeq2 (v1.30.1), comparing the two conditions (vehicle control and trametinib treated). Principal component analysis (PCA) was performed using the DESeq2 plotPCA function, and hierarchical clustering was run using Euclidian distance; both using rlog-transformed count data. Expression differences between control and trametinib treated samples were calculated using the DESeq results function with an alpha value of 0.05, and estimated fold changes were shrunk with the lfcShrink function using the apeglm method. Lists of significantly differentially expressed (DE) genes were identified using a *q* value (Benjamini-Hochberg corrected *p* value) cut-off of 0.05, (corresponding to a *p* value of ~0.00025).

Reads from control and trametinib treated tumorsphere RNA-seq libraries (3 days and 7 days post treatment) were similarly quantified with salmon (v.1.7.0) against an index built from the GENCODE v35 assembly with genomic decoy sequences. Data from both timepoints and both controls (15 samples) were loaded into R and filtered as described for the xenograft samples. Expression differences were calculated between trametinib treated samples and their matched controls at each time point, again as described for the xenograft libraries.

### Gene set enrichment analysis

GSEA (v4.1.0) was run using pre-ranked gene lists (the GSEA_preranked method) to explore enrichment of pathways and functional classes in genes expression changes following trametinib treatment of xenografts and tumorspheres. The DE gene lists were filtered to retain protein-coding genes, and ranked by −log10(p value) * sign(fold change). GSEA was run with the MSigDb (v7.4) collections H (hallmark gene sets), C2:CP (canonical pathways), C4:CGN (cancer gene networks), C4:CM (cancer modules), C6 (oncogenic signatures), and C8 (cell type signatures). Enrichment maps were generated in Cytoscape (v3.8.2) from GSEA results, following the protocol described by Reimand et al. [55]. Enriched gene sets with a *q* value < 0.005 were retained and clustered using the AutoAnnotate tool; figures were edited to remove unclustered gene sets, and small clusters unrelated to the cell types being studied.

### RNA-sequencing on mouse SHH MB trametinib treated cells

mRNA was purified from total RNA using poly-T oligo-attached magnetic beads. First strand cDNA was synthesized using random hexamer primers after fragmentation, followed by second strand cDNA synthesis using either dTTP for non-directional library or dUTP for directional library. Generated libraries were checked with Qubit and real-time PCR for quantification and the bioanalyzer for size distribution detection. Libraries were sequenced on the Illumina platform and paired-end reads were generated. Raw data (raw reads) in fastq format were processed through fastp software. Differential expression analysis of two conditions/group (two biological replicates per condition) was performed using the DESeq2 R package (1.20.0). The resulting *P* values were adjusted using the Benjamini-Hochberg approach. Genes with an adjusted *P* value ≤ 0.05 found by DESeq2 were differentially expressed. Pathway enrichment analysis of significant differentially expressed genes was conducted using g:profiler and comprised pathways from Reactome and GO Biological Processes (GO:BP). Enrichments were visualized using Cytoscape (v.3.7.1) and the Enrichment Map plug-in (v.3.5.1) with parameters FDR *Q* value < 0.001.

### Immunohistochemistry

Tumors from UI226, RCMB18 and HDMB03 xenografts were sectioned following formalin fixation and paraffin embedding. Slides were de-paraffinized and re-hydrated in xylene and ethanol gradients followed by antigen retrieval in sodium citrate buffer at 95–100 °C for 20 min. Slides were washed in 1× PBS and then treated for endogenous peroxidases for 10 min and again washed in 1×PBS. The samples were blocked in 3% sheep serum for 30 min at room temperature. They were then incubated with primary antibodies (Supplementary Table 1) prepared in 1% sheep serum/1× PBS overnight at 4 °C. The following day, the slides were first incubated in biotinylated secondary antibodies (Supplementary Table 1) for 2 h at room temperature. Streptavidin/HRP antibody (Jackson ImmunoResearch, West Grove, PA, USA) in 1× PBS was then added for 30 min at room temperature. Slides were then developed with DAB substrate (Sigma-Aldrich, St. Louis, Missouri, USA), counterstained with hematoxylin and mounted in Permount (ThermoFisher Scientific, Waltham, Massachusetts, USA).

### Statistical analysis

Data from in vitro and in vivo experiments were analyzed using Prism 9.0 software (GraphPad Software). Xenograft survival data were evaluated using the log-rank (Mantel–Cox) test. Tumorsphere size analysis was performed using two-sample Kolmogorov–Smirnov tests. One-way ANOVA followed by a Dunnett’s test for multiple comparisons were used to assess total tumorsphere number, cell viability, and cell migration. Homogeneity of variances was assessed using a Brown–Forsythe test. All data are reported as ±SEM. *P* values < 0.05 were considered significant. All human cell assays were performed using a minimum of three biological replicates and included technical replicates within each experiment to assess reproducibility.

### Supplementary information

Authorship Change Approval

Supplementary Legends and Figures

Original Data File

Supplementary Datasets

Supplementary Table - antibodies

## Data Availability

Human RNA-seq data can be accessed from GEO under GSE227921. The raw mouse RNA-seq data can be accessed through the following link: https://data.mendeley.com/datasets/7pwy8n4r6j/1.

## Code availability

R scripts for the analysis of tumorsphere and xenograft RNA-seq data are available in GitLab at https://gitlab.com/ohri/trametinib-treatment-of-shh-medulloblastoma.

## References

[1] Hovestadt V, Ayrault O, Swartling FJ, Robinson GW, Pfister SM, Northcott PA (2020). Medulloblastomics revisited: biological and clinical insights from thousands of patients. Nat Rev Cancer.

[2] Mehta M, Chang S, Newton H, Guha A, Vogelbaum M. Principles and Practice of Neuro-oncology: a multidisciplinary approach: 1 edition. (Demos Medical Publishing, New York, 2011).

[3] Taylor MD, Northcott PA, Korshunov A, Remke M, Cho YJ, Clifford SC (2012). Molecular subgroups of medulloblastoma: the current consensus. Acta Neuropathol.

[4] Louis DN, Perry A, Wesseling P, Brat DJ, Cree IA, Figarella-Branger D (2021). The 2021 WHO Classification of Tumors of the Central Nervous System: a summary. Neuro Oncol.

[5] Northcott PA, Korshunov A, Witt H, Hielscher T, Eberhart CG, Mack S (2011). Medulloblastoma comprises four distinct molecular variants. J Clin Oncol : Off J Am Soc Clin Oncol.

[6] Gajjar A, Robinson GW, Smith KS, Lin T, Merchant TE, Chintagumpala M (2021). Outcomes by Clinical and Molecular Features in Children With Medulloblastoma Treated With Risk-Adapted Therapy: Results of an International Phase III Trial (SJMB03). J Clin Oncol: Off J Am Soc Clin Oncol.

[7] Cavalli FMG, Remke M, Rampasek L, Peacock J, Shih DJH, Luu B (2017). Intertumoral Heterogeneity within Medulloblastoma Subgroups. Cancer Cell.

[8] Schwalbe EC, Lindsey JC, Nakjang S, Crosier S, Smith AJ, Hicks D (2017). Novel molecular subgroups for clinical classification and outcome prediction in childhood medulloblastoma: a cohort study. Lancet Oncol.

[9] Zhukova N, Ramaswamy V, Remke M, Pfaff E, Shih DJ, Martin DC (2013). Subgroup-specific prognostic implications of TP53 mutation in medulloblastoma. J Clin Oncol : Off J Am Soc Clin Oncol.

[10] Ramaswamy V, Remke M, Adamski J, Bartels U, Tabori U, Wang X (2016). Medulloblastoma subgroup-specific outcomes in irradiated children: who are the true high-risk patients?. Neuro Oncol.

[11] Ramaswamy V, Remke M, Bouffet E, Bailey S, Clifford SC, Doz F (2016). Risk stratification of childhood medulloblastoma in the molecular era: the current consensus. Acta Neuropathol.

[12] Robinson GW, Rudneva VA, Buchhalter I, Billups CA, Waszak SM, Smith KS (2018). Risk-adapted therapy for young children with medulloblastoma (SJYC07): therapeutic and molecular outcomes from a multicentre, phase 2 trial. Lancet Oncol.

[13] Garcia-Lopez J, Kumar R, Smith KS, Northcott PA (2021). Deconstructing Sonic Hedgehog Medulloblastoma: Molecular Subtypes, Drivers, and Beyond. Trends Genet.

[14] WHO Classification of Tumours Editorial Board. World Health Organization Classification of Tumours of the Central Nervous System, 5th Edition. (International Agency for Research on Cancer, Lyon, 2021).

[15] Yauch RL, Dijkgraaf GJ, Alicke B, Januario T, Ahn CP, Holcomb T (2009). Smoothened mutation confers resistance to a Hedgehog pathway inhibitor in medulloblastoma. Science.

[16] Dijkgraaf GJ, Alicke B, Weinmann L, Januario T, West K, Modrusan Z (2011). Small molecule inhibition of GDC-0449 refractory smoothened mutants and downstream mechanisms of drug resistance. Cancer Res.

[17] Robinson GW, Orr BA, Wu G, Gururangan S, Lin T, Qaddoumi I (2015). Vismodegib Exerts Targeted Efficacy Against Recurrent Sonic Hedgehog-Subgroup Medulloblastoma: Results From Phase II Pediatric Brain Tumor Consortium Studies PBTC-025B and PBTC-032. J Clin Oncol:Off J Am Soc Clin Oncol.

[18] Kool M, Jones DT, Jager N, Northcott PA, Pugh TJ, Hovestadt V (2014). Genome sequencing of SHH medulloblastoma predicts genotype-related response to smoothened inhibition. Cancer Cell.

[19] Liang L, Coudiere-Morrison L, Tatari N, Stromecki M, Fresnoza A, Porter CJ (2018). CD271(+) Cells Are Diagnostic and Prognostic and Exhibit Elevated MAPK Activity in SHH Medulloblastoma. Cancer Res.

[20] Liang L, Aiken C, McClelland R, Morrison LC, Tatari N, Remke M (2015). Characterization of novel biomarkers in selecting for subtype specific medulloblastoma phenotypes. Oncotarget.

[21] Dombi E, Baldwin A, Marcus LJ, Fisher MJ, Weiss B, Kim A (2016). Activity of Selumetinib in Neurofibromatosis Type 1-Related Plexiform Neurofibromas. N Engl J Med.

[22] Gross A, Bishop R, Widemann BC (2017). Selumetinib in Plexiform Neurofibromas. N Engl J Med.

[23] Banerjee A, Jakacki RI, Onar-Thomas A, Wu S, Nicolaides T, Young Poussaint T (2017). A phase I trial of the MEK inhibitor selumetinib (AZD6244) in pediatric patients with recurrent or refractory low-grade glioma: a Pediatric Brain Tumor Consortium (PBTC) study. Neuro Oncol.

[24] Fangusaro J, Onar-Thomas A, Young Poussaint T, Wu S, Ligon AH, Lindeman N (2019). Selumetinib in paediatric patients with BRAF-aberrant or neurofibromatosis type 1-associated recurrent, refractory, or progressive low-grade glioma: a multicentre, phase 2 trial. Lancet Oncol.

[25] Wu PK, Park JI (2015). MEK1/2 Inhibitors: Molecular Activity and Resistance Mechanisms. Semin Oncol.

[26] Perreault S, Larouche V, Tabori U, Hawkin C, Lippe S, Ellezam B (2019). A phase 2 study of trametinib for patients with pediatric glioma or plexiform neurofibroma with refractory tumor and activation of the MAPK/ERK pathway: TRAM-01. BMC Cancer.

[27] Bouffet E, Kiernan M, Hargrave D, Roberts S, Aerts I, Broniscer A (2018). Trametinib therapy in pediatric patients with low-grade gliomas (LGG) with BRAF gene fusion;a disease specific cohort in the first pediatric testing of trametinib. Neuro-Oncol.

[28] Manoharan N, Choi J, Chordas C, Zimmerman MA, Scully J, Clymer J (2020). Trametinib for the treatment of recurrent/progressive pediatric low-grade glioma. J Neurooncol.

[29] Selt F, van Tilburg CM, Bison B, Sievers P, Harting I, Ecker J (2020). Response to trametinib treatment in progressive pediatric low-grade glioma patients. J Neurooncol.

[30] Werbowetski-Ogilvie TE, Morrison LC, Fiebig-Comyn A, Bhatia M (2012). In vivo generation of neural tumors from neoplastic pluripotent stem cells models early human pediatric brain tumor formation. Stem Cells.

[31] Lee J, Kotliarova S, Kotliarov Y, Li A, Su Q, Donin NM (2006). Tumor stem cells derived from glioblastomas cultured in bFGF and EGF more closely mirror the phenotype and genotype of primary tumors than do serum-cultured cell lines. Cancer Cell.

[32] Zagozewski J, Shahriary GM, Morrison LC, Saulnier O, Stromecki M, Fresnoza A (2020). An OTX2-PAX3 signaling axis regulates Group 3 medulloblastoma cell fate. Nat Commun.

[33] Markowitz D, Powell C, Tran NL, Berens ME, Ryken TC, Vanan M (2016). Pharmacological Inhibition of the Protein Kinase MRK/ZAK Radiosensitizes Medulloblastoma. Mol Cancer Ther.

[34] Northcott PA, Shih DJ, Remke M, Cho YJ, Kool M, Hawkins C (2012). Rapid, reliable, and reproducible molecular sub-grouping of clinical medulloblastoma samples. Acta Neuropathol.

[35] Jacobsen PF, Jenkyn DJ, Papadimitriou JM (1985). Establishment of a human medulloblastoma cell line and its heterotransplantation into nude mice. J Neuropathol Exp Neurol.

[36] Ward RJ, Lee L, Graham K, Satkunendran T, Yoshikawa K, Ling E (2009). Multipotent CD15+ cancer stem cells in patched-1-deficient mouse medulloblastoma. Cancer Res.

[37] Zhao X, Ponomaryov T, Ornell KJ, Zhou P, Dabral SK, Pak E (2015). RAS/MAPK Activation Drives Resistance to Smo Inhibition, Metastasis, and Tumor Evolution in Shh Pathway-Dependent Tumors. Cancer Res.

[38] Rusert JM, Juarez EF, Brabetz S, Jensen J, Garancher A, Chau LQ (2020). Functional precision medicine identifies new therapeutic candidates for medulloblastoma. Cancer Res.

[39] Gao M, Yang J, Gong H, Lin Y, Liu J (2021). Trametinib Inhibits the Growth and Aerobic Glycolysis of Glioma Cells by Targeting the PKM2/c-Myc Axis. Front Pharm.

[40] Zagozewski J, Borlase S, Guppy BJ, Coudiere-Morrison L, Shahriary GM, Gordon V (2022). Combined MEK and JAK/STAT3 pathway inhibition effectively decreases SHH medulloblastoma tumor progression. Commun Biol.

[41] Bangs F, Anderson KV (2017). Primary Cilia and Mammalian Hedgehog Signaling. Cold Spring Harb Perspect Biol.

[42] Chang CH, Zanini M, Shirvani H, Cheng JS, Yu H, Feng CH (2019). Atoh1 Controls Primary Cilia Formation to Allow for SHH-Triggered Granule Neuron Progenitor Proliferation. Dev Cell.

[43] Chizhikov VV, Davenport J, Zhang Q, Shih EK, Cabello OA, Fuchs JL (2007). Cilia proteins control cerebellar morphogenesis by promoting expansion of the granule progenitor pool. J Neurosci.

[44] Spassky N, Han YG, Aguilar A, Strehl L, Besse L, Laclef C (2008). Primary cilia are required for cerebellar development and Shh-dependent expansion of progenitor pool. Dev Biol.

[45] Han YG, Kim HJ, Dlugosz AA, Ellison DW, Gilbertson RJ, Alvarez-Buylla A (2009). Dual and opposing roles of primary cilia in medulloblastoma development. Nat Med.

[46] Zhao X, Pak E, Ornell KJ, Pazyra-Murphy MF, MacKenzie EL, Chadwick EJ (2017). A Transposon Screen Identifies Loss of Primary Cilia as a Mechanism of Resistance to SMO Inhibitors. Cancer Discov.

[47] Kuonen F, Huskey NE, Shankar G, Jaju P, Whitson RJ, Rieger KE (2019). Loss of Primary Cilia Drives Switching from Hedgehog to Ras/MAPK Pathway in Resistant Basal Cell Carcinoma. J Investig Dermatol.

[48] Morrison LC, McClelland R, Aiken C, Bridges M, Liang L, Wang X (2013). Deconstruction of medulloblastoma cellular heterogeneity reveals differences between the most highly invasive and self-renewing phenotypes. Neoplasia.

[49] Kaur R, Aiken C, Morrison LC, Rao R, Del Bigio MR, Rampalli S (2015). OTX2 exhibits cell-context-dependent effects on cellular and molecular properties of human embryonic neural precursors and medulloblastoma cells. Dis Model Mech.

[50] Morfouace M, Shelat A, Jacus M, Freeman BB, Turner D, Robinson S (2014). Pemetrexed and gemcitabine as combination therapy for the treatment of Group3 medulloblastoma. Cancer Cell.

[51] Milde T, Lodrini M, Savelyeva L, Korshunov A, Kool M, Brueckner LM (2012). HD-MB03 is a novel Group 3 medulloblastoma model demonstrating sensitivity to histone deacetylase inhibitor treatment. J Neurooncol.

[52] Cancer M, Hutter S, Holmberg KO, Rosen G, Sundstrom A, Tailor J (2019). Humanized Stem Cell Models of Pediatric Medulloblastoma Reveal an Oct4/mTOR Axis that Promotes Malignancy. Cell Stem Cell.

[53] Kluin RJC, Kemper K, Kuilman T, de Ruiter JR, Iyer V, Forment JV (2018). XenofilteR: computational deconvolution of mouse and human reads in tumor xenograft sequence data. BMC Bioinforma.

[54] Patro R, Duggal G, Love MI, Irizarry RA, Kingsford C (2017). Salmon provides fast and bias-aware quantification of transcript expression. Nat Methods.

[55] Reimand J, Isserlin R, Voisin V, Kucera M, Tannus-Lopes C, Rostamianfar A (2019). Pathway enrichment analysis and visualization of omics data using g:Profiler, GSEA, Cytoscape and EnrichmentMap. Nat Protoc.

