# Supplementary material for: Cross-species analysis of SHH medulloblastoma models reveals significant inhibitory effects of trametinib on tumor progression
Source: Cell Death Discov. 2023 Sep 19;9:347. doi: 10.1038/s41420-023-01646-0 (PMC10509237; doi:10.1038/s41420-023-01646-0)
Supplement: Supplementary file 1 — Authorship Change Approval [file 41420_2023_1646_MOESM1_ESM.pdf]

#1 Borlase Approval

Subject: Re: CDDISCOVERY-23-1272R1 Initial Quality Check
Date: Wednesday, August 30, 2023 at 8:04:38 AM Central Daylight Time
From: Stephanie Borlase
To: Ogilvie, Tamra Erin
CC: Ogilvie, Tamra Erin, cporter@ohri.ca, Vijay Ramaswamy, Lisa.Liang@umanitoba.ca, Alexandria DeCarlo

Hi Tamra,

I agree.

Best,
Steph

On Aug 30, 2023, at 8:36 AM, Alexandria DeCarlo <alexandria.decarlo@sickkids.ca> wrote:

> You don't often get email from alexandria.decarlo@sickkids.ca. Learn why this is important

Hi Tamra,

I agree

Alexandria DeCarlo
PhD Student | Ramaswamy Lab
Department of Medical Biophysics | University of Toronto
SickKids | Peter Gilgan Centre for Research and Learning
686 Bay Street, Toronto, ON

From: Ogilvie, Tamra Erin <Tamra.Ogilvie@bcm.edu>
Sent: August 30, 2023 7:44 AM
To: cporter@ohri.ca <cporter@ohri.ca>; Vijay Ramaswamy <vijay.ramaswamy@sickkids.ca>; Alexandria DeCarlo <alexandria.decarlo@sickkids.ca>; Stephanie Borlase <stephanie.borlase@mail.utoronto.ca>
Cc: Lisa.Liang@umanitoba.ca <Lisa.Liang@umanitoba.ca>
Subject: Fw: CDDISCOVERY-23-1272R1 Initial Quality Check

Hi everyone, my PDF Lisa Liang, was added as a co-author to the paper. Since this happened after the original submission, the journal CDD requires that I seek approval from all the authors before they can formally accept the paper.

#2 Del Carlo Approval

Subject: Re: CDDISCOVERY-23-1272R1 Initial Quality Check
Date: Wednesday, August 30, 2023 at 7:36:30 AM Central Daylight Time
From: Alexandria DeCarlo
To: Ogilvie, Tamra Erin, cporter@ohri.ca, Vijay Ramaswamy, Stephanie Borlase
CC: Lisa.Liang@umanitoba.ca

Hi Tamra,

I agree

Alexandria DeCarlo
PhD Student | Ramaswamy Lab
Department of Medical Biophysics | University of Toronto
SickKids | Peter Gilgan Centre for Research and Learning
686 Bay Street, Toronto, ON

From: Ogilvie, Tamra Erin <Tamra.Ogilvie@bcm.edu>
Sent: August 30, 2023 7:44 AM
To: cporter@ohri.ca <cporter@ohri.ca>; Vijay Ramaswamy <vijay.ramaswamy@sickkids.ca>; Alexandria DeCarlo <alexandria.decarlo@sickkids.ca>; Stephanie Borlase <stephanie.borlase@mail.utoronto.ca>
Cc: Lisa.Liang@umanitoba.ca <Lisa.Liang@umanitoba.ca>
Subject: Fw: CDDISCOVERY-23-1272R1 Initial Quality Check

Hi everyone, my PDF Lisa Liang, was added as a co-author to the paper. Since this happened after the original submission, the journal CDD requires that I seek approval from all the authors before they can formally accept the paper.

They just need each of you to reply to the email confirming that you accept the change. I will need to pool the answers and upload to the journal.

Thanks!
Tamra

Dr. Tamra Ogilvie
Department of Pediatrics, Section of Hematology-Oncology
Baylor College of Medicine
Texas Children's Hospital
Feigin Tower
1102 Bates Ave., Suite C.1030.15
Houston, TX
77030
Phone: 832-824-5667
Website: https://www.texaschildrens.org/find-a-doctor/tamra-e-ogilvie-phd

#3 Porter Approval

Subject: Re: CDDISCOVERY-23-1272R1 Initial Quality Check
Date: Wednesday, August 30, 2023 at 6:54:55 AM Central Daylight Time
From: Christopher Porter
To: Ogilvie, Tamra Erin, Vijay Ramaswamy, Alexandria DeCarlo, Stephanie Borlase
CC: Lisa.Liang@umanitoba.ca

Hi Tamra,

I agree to adding Lisa Liang as an co-author of the paper "Cross-species analysis of SHH medulloblastoma models reveals significant inhibitory effects of trametinib on tumor progression".

Chris

Christopher Porter
Bioinformaticist | Bio-informaticist
Ottawa Bioinformatics Core Facility
Sprott Centre for Stem Cell Research
Ottawa Hospital Research Institute | Institut de la recherche de l'Hôpital d'Ottawa
T: 613-737-8899, 73255

From: Ogilvie, Tamra Erin <Tamra.Ogilvie@bcm.edu>
Date: Wednesday, August 30, 2023 at 7:44 AM
To: Christopher Porter <cporter@ohri.ca>, Vijay Ramaswamy <vijay.ramaswamy@sickkids.ca>, Alexandria DeCarlo <alexandria.decarlo@sickkids.ca>, Stephanie Borlase <stephanie.borlase@mail.utoronto.ca>
Cc: Lisa.Liang@umanitoba.ca <Lisa.Liang@umanitoba.ca>
Subject: Fw: CDDISCOVERY-23-1272R1 Initial Quality Check

CAUTION: External Mail. Do not click on links or open attachments you do not trust.
ATTENTION: Courriel externe. Ne cliquez pas sur des liens et n'ouvrez pas de pièces jointes auxquelles vous ne faites pas confiance.

Hi everyone, my PDF Lisa Liang, was added as a co-author to the paper. Since this happened after the original submission, the journal CDD requires that I seek approval from all the authors before they can formally accept the paper.

They just need each of you to reply to the email confirming that you accept the change. I will need to pool the answers and upload to the journal.

Thanks!
Tamra

Dr. Tamra Ogilvie

Department of Pediatrics, Section of Hematology-Oncology

Baylor College of Medicine

Texas Children's Hospital

Feigin Tower

1102 Bates Ave., Suite C.1030.15

Houston, TX

77030

#4 Ramaswamy Approval

Subject: Re: CDDISCOVERY-23-1272R1 Initial Quality Check
Date: Wednesday, August 30, 2023 at 7:27:20 AM Central Daylight Time
From: Vijay Ramaswamy
To: Ogilvie, Tamra Erin, cporter@ohri.ca, Alexandria DeCarlo, Stephanie Borlase
CC: Lisa.Liang@umanitoba.ca

I accept

Sent from my iPhone

From: Ogilvie, Tamra Erin <Tamra.Ogilvie@bcm.edu>
Sent: Wednesday, August 30, 2023 7:44:13 AM
To: cporter@ohri.ca <cporter@ohri.ca>; Vijay Ramaswamy <vijay.ramaswamy@sickkids.ca>; Alexandria DeCarlo <alexandria.decarlo@sickkids.ca>; Stephanie Borlase <stephanie.borlase@mail.utoronto.ca>
Cc: Lisa.Liang@umanitoba.ca <Lisa.Liang@umanitoba.ca>
Subject: Fw: CDDISCOVERY-23-1272R1 Initial Quality Check

Hi everyone, my PDF Lisa Liang, was added as a co-author to the paper. Since this happened after the original submission, the journal CDD requires that I seek approval from all the authors before they can formally accept the paper.

They just need each of you to reply to the email confirming that you accept the change. I will need to pool the answers and upload to the journal.

Thanks!
Tamra

Dr. Tamra Ogilvie
Department of Pediatrics, Section of Hematology-Oncology
Baylor College of Medicine
Texas Children's Hospital
Feigin Tower
1102 Bates Ave., Suite C.1030.15
Houston, TX
77030
Phone: 832-824-5667
Website: https://www.texaschildrens.org/find-a-doctor/tamra-e-ogilvie-phd
Twitter: @OgilvieTamra

From: cddiscovery@springernature.com <cddiscovery@springernature.com>
Sent: August 30, 2023 6:31 AM
To: Ogilvie, Tamra Erin <Tamra.Ogilvie@bcm.edu>
Subject: CDDISCOVERY-23-1272R1 Initial Quality Check

#5 Morrison Approval

Subject: Re: CDDISCOVERY-23-1272R1 Initial Quality Check
Date: Wednesday, August 30, 2023 at 8:53:41 AM Central Daylight Time
From: Ludivine Morrison
To: Ogilvie, Tamra Erin

Hi Tamra,
I accept to add Lisa Liang as a co-author to the paper.

Ludivine M.C. Morrison

Sent from my iPhone

On Aug 30, 2023, at 8:39 AM, Ogilvie, Tamra Erin <Tamra.Ogilvie@bcm.edu> wrote:

Hi Lu! Please see email below and respond whether you accept Lisa as an author.

Thanks!
Tamra

From: Ogilvie, Tamra Erin <Tamra.Ogilvie@bcm.edu>
Date: Wednesday, August 30, 2023 at 6:44 AM
To: cporter@ohri.ca <cporter@ohri.ca>, Vijay Ramaswamy <vijay.ramaswamy@sickkids.ca>, Alexandria DeCarlo <alexandria.decarlo@sickkids.ca>, Stephanie Borlase <stephanie.borlase@mail.utoronto.ca>
Cc: Lisa.Liang@umanitoba.ca <Lisa.Liang@umanitoba.ca>
Subject: Fw: CDDISCOVERY-23-1272R1 Initial Quality Check

Hi everyone, my PDF Lisa Liang, was added as a co-author to the paper. Since this happened after the original submission, the journal CDD requires that I seek approval from all the authors before they can formally accept the paper.

They just need each of you to reply to the email confirming that you accept the change. I will need to pool the answers and upload to the journal.

Thanks!
Tamra

Dr. Tamra Ogilvie

#6 Liang Approval

Subject: Re: CDDISCOVERY-23-1272R1 Initial Quality Check
Date: Wednesday, August 30, 2023 at 9:16:30 AM Central Daylight Time
From: Lisa Liang
To: Stephanie Borlase, Ogilvie, Tamra Erin
CC: Ogilvie, Tamra Erin, cporter@ohri.ca, Vijay Ramaswamy, Alexandria DeCarlo

Hi Tamra,

I accept.

Thanks,
Lisa

Get Outlook for iOS

From: Stephanie Borlase <stephanie.borlase@mail.utoronto.ca>
Sent: Wednesday, August 30, 2023 8:04:00 AM
To: Tamra Erin Ogilvie <Tamra.Ogilvie@bcm.edu>
Cc: Ogilvie, Tamra Erin <Tamra.Ogilvie@bcm.edu>; cporter@ohri.ca <cporter@ohri.ca>; Vijay Ramaswamy <vijay.ramaswamy@sickkids.ca>; Lisa Liang <Lisa.Liang@umanitoba.ca>; Alexandria DeCarlo <alexandria.decarlo@sickkids.ca>
Subject: Re: CDDISCOVERY-23-1272R1 Initial Quality Check

Caution: This message was sent from outside the University of Manitoba.

Hi Tamra,

I agree.

Best,
Steph

> On Aug 30, 2023, at 8:36 AM, Alexandria DeCarlo <alexandria.decarlo@sickkids.ca> wrote:
>
> You don't often get email from alexandria.decarlo@sickkids.ca. Learn why this is important
>
> Hi Tamra,
>
> I agree

#7 Werbowetski-Ogilvie Approval

Subject: Re: CDDISCOVERY-23-1272R1 Initial Quality Check
Date: Wednesday, August 30, 2023 at 9:17:45 AM Central Daylight Time
From: Ogilvie, Tamra Erin
To: Lisa Liang, Stephanie Borlase
CC: cporter@ohri.ca, Vijay Ramaswamy, Alexandria DeCarlo

Thanks to everyone for the quick responses.

I also accept the addition of Lisa as a co-author.

Tamra

From: Lisa Liang <Lisa.Liang@umanitoba.ca>
Date: Wednesday, August 30, 2023 at 9:16 AM
To: Stephanie Borlase <stephanie.borlase@mail.utoronto.ca>, Ogilvie, Tamra Erin <Tamra.Ogilvie@bcm.edu>
Cc: Ogilvie, Tamra Erin <Tamra.Ogilvie@bcm.edu>, cporter@ohri.ca <cporter@ohri.ca>, Vijay Ramaswamy <vijay.ramaswamy@sickkids.ca>, Alexandria DeCarlo <alexandria.decarlo@sickkids.ca>
Subject: Re: CDDISCOVERY-23-1272R1 Initial Quality Check

Hi Tamra,

I accept.

Thanks,
Lisa

Get Outlook for iOS

From: Stephanie Borlase <stephanie.borlase@mail.utoronto.ca>
Sent: Wednesday, August 30, 2023 8:04:00 AM
To: Tamra Erin Ogilvie <Tamra.Ogilvie@bcm.edu>
Cc: Ogilvie, Tamra Erin <Tamra.Ogilvie@bcm.edu>; cporter@ohri.ca <cporter@ohri.ca>; Vijay Ramaswamy <vijay.ramaswamy@sickkids.ca>; Lisa Liang <Lisa.Liang@umanitoba.ca>; Alexandria DeCarlo <alexandria.decarlo@sickkids.ca>
Subject: Re: CDDISCOVERY-23-1272R1 Initial Quality Check

Caution: This message was sent from outside the University of Manitoba.

Hi Tamra,

I agree.

Best,
Steph

On Aug 30, 2023, at 8:36 AM, Alexandria DeCarlo <alexandria.decarlo@sickkids.ca> wrote:

You don't often get email from alexandria.decarlo@sickkids.ca. Learn why this is important

Hi Tamra,

I agree

Alexandria DeCarlo
PhD Student I Ramaswamy Lab
Department of Medical Biophysics I University of Toronto
SickKids I Peter Gilgan Centre for Research and Learning
686 Bay Street, Toronto, ON

From: Ogilvie, Tamra Erin <Tamra.Ogilvie@bcm.edu>
Sent: August 30, 2023 7:44 AM
To: cporter@ohri.ca <cporter@ohri.ca>; Vijay Ramaswamy <vijay.ramaswamy@sickkids.ca>; Alexandria DeCarlo <alexandria.decarlo@sickkids.ca>; Stephanie Borlase <stephanie.borlase@mail.utoronto.ca>
Cc: Lisa.Liang@umanitoba.ca <Lisa.Liang@umanitoba.ca>
Subject: Fw: CDDISCOVERY-23-1272R1 Initial Quality Check

Hi everyone, my PDF Lisa Liang, was added as a co-author to the paper. Since this happened after the original submission, the journal CDD requires that I seek approval from all the authors before they can formally accept the paper.

They just need each of you to reply to the email confirming that you accept the change. I will need to pool the answers and upload to the journal.

Thanks!
Tamra

Dr. Tamra Ogilvie
Department of Pediatrics, Section of Hematology-Oncology
Baylor College of Medicine
Texas Children's Hospital
Feigin Tower
1102 Bates Ave., Suite C.1030.15
Houston, TX
77030
Phone: 832-824-5667
Website: https://www.texaschildrens.org/find-a-doctor/tamra-e-ogilvie-phd
