# Supplementary material for: Cross-species analysis of SHH medulloblastoma models reveals significant inhibitory effects of trametinib on tumor progression
Source: Cell Death Discov. 2023 Sep 19;9:347. doi: 10.1038/s41420-023-01646-0 (PMC10509237; doi:10.1038/s41420-023-01646-0)
Supplement: Supplementary file 2 — Supplementary Legends and Figures [file 41420_2023_1646_MOESM2_ESM.pdf]

Cross-species analysis of SHH medulloblastoma models reveals significant inhibitory effects of trametinib on tumor progression

Stephanie Borlase, Alexandria DeCarlo, Ludivine Coudière-Morrison, Lisa Liang, Christopher J. Porter, Vijay Ramaswamy, Tamra E. Werbowetski-Ogilvie

Supplementary Figures and Legends

Supplementary Figure 1. Treatment with the MEKi inhibitor selumetinib or trametinib significantly impedes Daoy SHH MB tumorigenic properties *in vitro*.

A-B. Western blot for p-ERK1/2, total ERK1/2 and GAPDH following treatment of Daoy SHH MB tumorspheres with (A) selumetinib or (B) trametinib for 3 days.

C-D. Representative images of Daoy tumorspheres following treatment with various concentrations of selumetinib (C) or trametinib (D). Scale bar, 400 μm.

E-F. Daoy tumorsphere size following treatment with increasing doses of selumetinib (E) or trametinib (F). Error bars: SEM. N=6 biological replicates and n=4 technical replicates for each biological replicate. Statistical analysis was completed using the Kolmogorov-Smirnov test. *, $P < 0.05$; **, $P < 0.01$; ***, $P < 0.001$; ****, $P < 0.0001$. For E: DMSO vs. 1 μM, p=0.0479; DMSO vs. 5μM, p=0.0044; DMSO vs. 10 μM, p=0.0002; DMSO vs. 20 μM, p<0.0001. For F: DMSO vs. 50 nM, p=0.0003; DMSO vs. 100 nM, p=0.0002; DMSO vs. 250 nM, 500 nM and 1μM, p<0.0001.

G-H. Daoy tumorsphere number following treatment with increasing doses of selumetinib (G) or trametinib (H). Error bars: SEM. N=6 biological replicates and n=4 technical replicates for each biological replicate. Statistical analysis was completed using the Dunnett's test for multiple

comparisons. *, $P < 0.05$; **, $P < 0.01$; ****, $P < 0.0001$. For H: DMSO vs. 250 nM, p=0.0442; DMSO vs. 500 nM, p=0.009, DMSO vs. 1 μM, p<0.0001.

I-J. Daoy cell viability following treatment with increasing doses of selumetinib (I) or trametinib (J). Error bars: SEM. N=6 biological replicates and n=4 technical replicates for each biological replicate. Statistical analysis was completed using the Dunnett's test for multiple comparisons. *, $P < 0.05$; **, $P < 0.01$; ***, $P < 0.001$; ****, $P < 0.0001$. For I: DMSO vs. 20 μM, p<0.0001. For J: DMSO vs. 100 nM, p=0.0349; DMSO vs. 250 nM, p=0.0095; DMSO vs. 500 nM, p=0.001; DMSO vs. 1 μM, p<0.0001.

K. Daoy migration following treatment with increasing doses of trametinib. Error bars: SEM. N=4 biological replicates and n=4 technical replicates for each biological replicate. Statistical analysis was completed using the Dunnett's test for multiple comparisons. *, $P < 0.05$; **, $P < 0.01$. DMSO vs. 500 nM, p=0.0244; DMSO vs. 1 μM, p=0.0029.

Supplementary Figure 2. Trametinib treatment results in significant changes in gene expression at both day 3 and day 7.

A. Principal component analysis (PCA) plotting principal components 1 and 2 for data from DMSO- and trametinib-treated UI226 tumorsphere RNA-seq libraries. PCA was run with the DESeq2 plotPCA function, using the 500 most variably expressed genes in the dataset.

B. Heatmap showing the expression of the most significantly differentially expressed genes in tumorsphere RNA-seq data following three- or seven-days (D3 or D7) treatment with trametinib. Normalized expression counts per replicate were taken for all genes with an adjusted p-value < 0.01 in the DESeq2 comparison of trametinib vs DMSO treated tumorspheres at either D3 or D7. The resulting expression matrix is displayed as a heatmap, showing the gene-wise Z-score. Both

rows (genes) and columns (replicates) were clustered using the R hclust function with Euclidian distance and using the complete agglomeration method.

Supplementary Figure 3. Trametinib significantly alters expression of cell cycle and differentiation genes.

A-D. GSEA depicting genes associated with E2F targets (A), Myc targets (B), G2M checkpoint (C), as well as neural stem cell markers (D) that are enriched in downregulated genes sets following 3 days of trametinib treatment. padj<0.0001 for all signatures except for C: q=0.00875 for G2M checkpoint.

E-H. GSEA depicting genes associated with E2F targets (E), Myc targets (F), G2M checkpoint (G), as well as neural stem cell markers (H) that are enriched in downregulated genes sets following 7 days of trametinib treatment. padj<0.0001 for all signatures.

I-K. Heat maps depicting genes associated with SHH signaling (I), astrocytic markers (J) and ciliopathy markers (K) that are either enriched in downregulated genes sets (I) or upregulated gene sets (J-K) following 7 days of trametinib treatment.

Supplementary Figure 4. pERK staining is variable across MB xenograft models and trametinib does not induce toxicity.

A. Representative images of immunohistochemical staining for p-ERK antibody in FFPE tissue sections derived from 3 representative independent control tumors from UI226 SHH MB, RCMB18 SHH MB and HDMB03 Group 3 MB xenografts. Scale bar: 150 µm.

B. NOD SCID mice without tumors were administered vehicle or 3 different concentrations of trametinib to evaluate drug toxicity over 28 days. Each treatment group consisted of 2 mice that

were administered 0.1 mL trametinib or vehicle control once daily via oral gavage on a 5 day on, 2 day off schedule. After 28 days, the animals showed no signs of toxicity and continuously gained weight. Error bars: SEM.

Borlase et al. Supplementary Figure 1

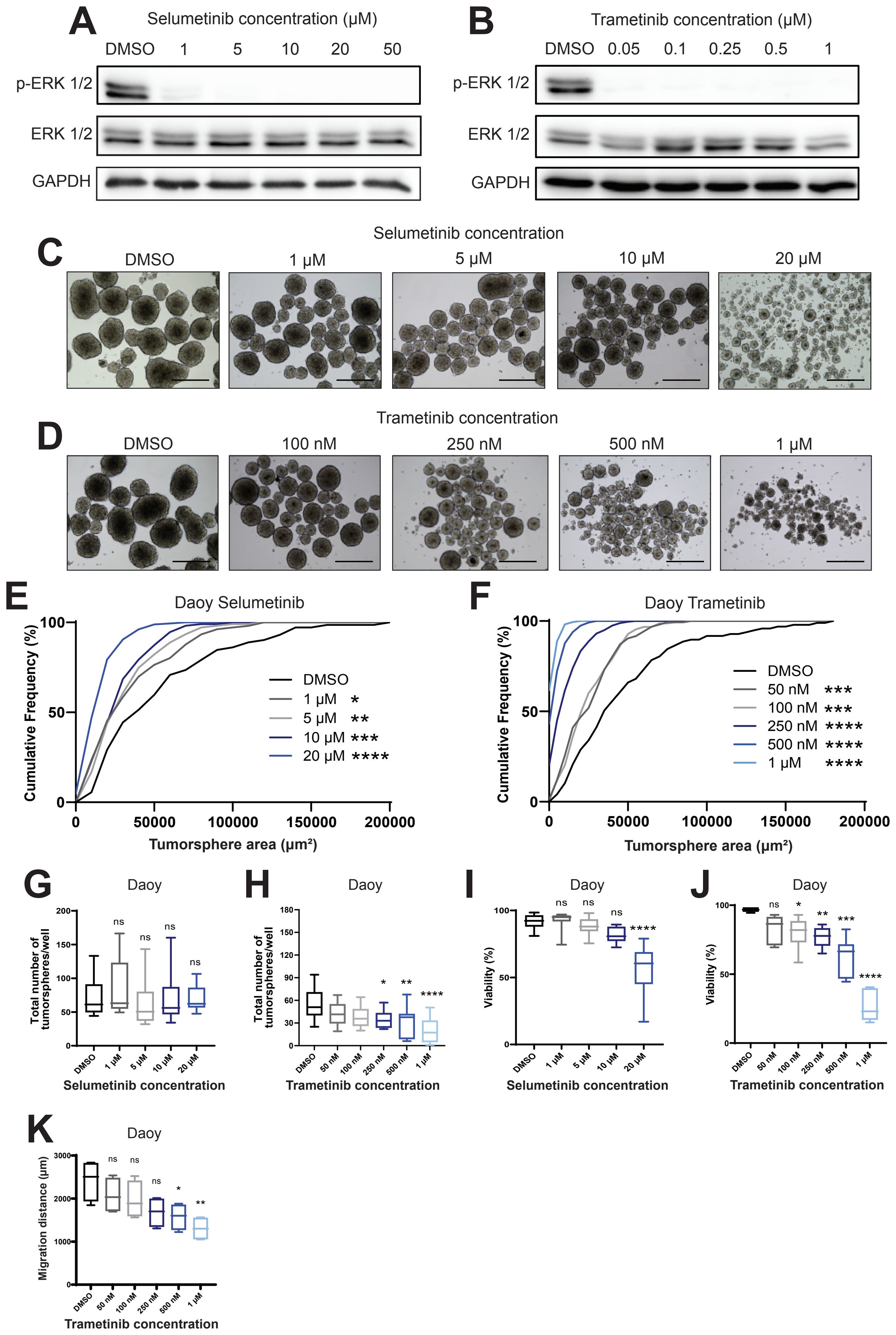

Borlase et al. Supplementary Figure 2

A

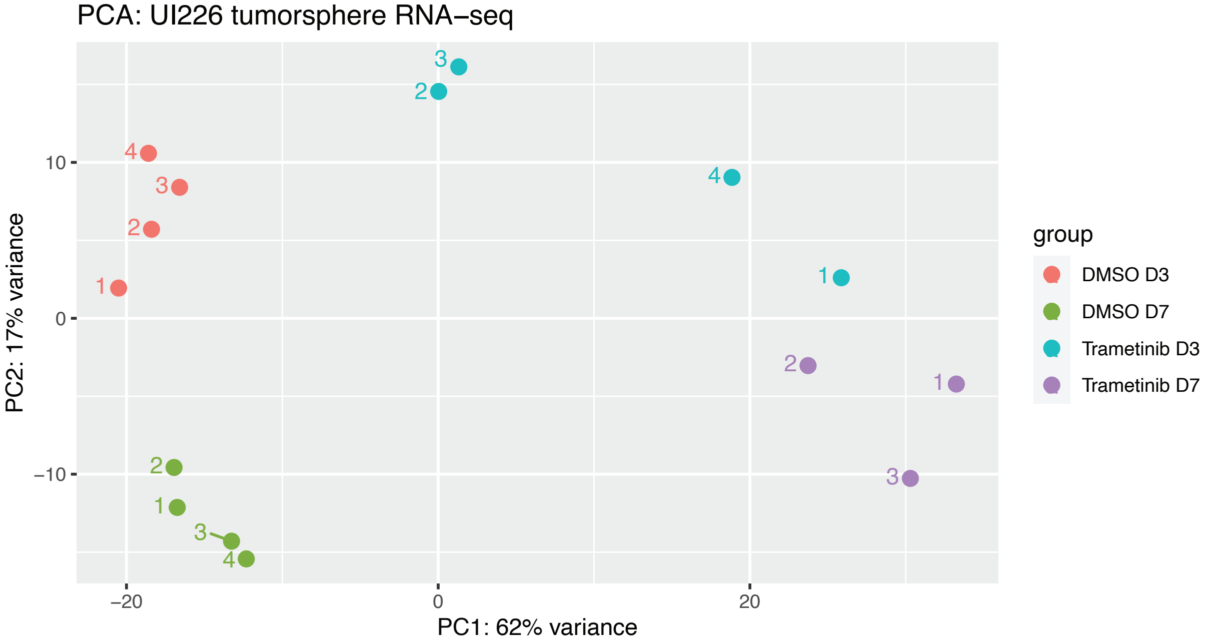

B

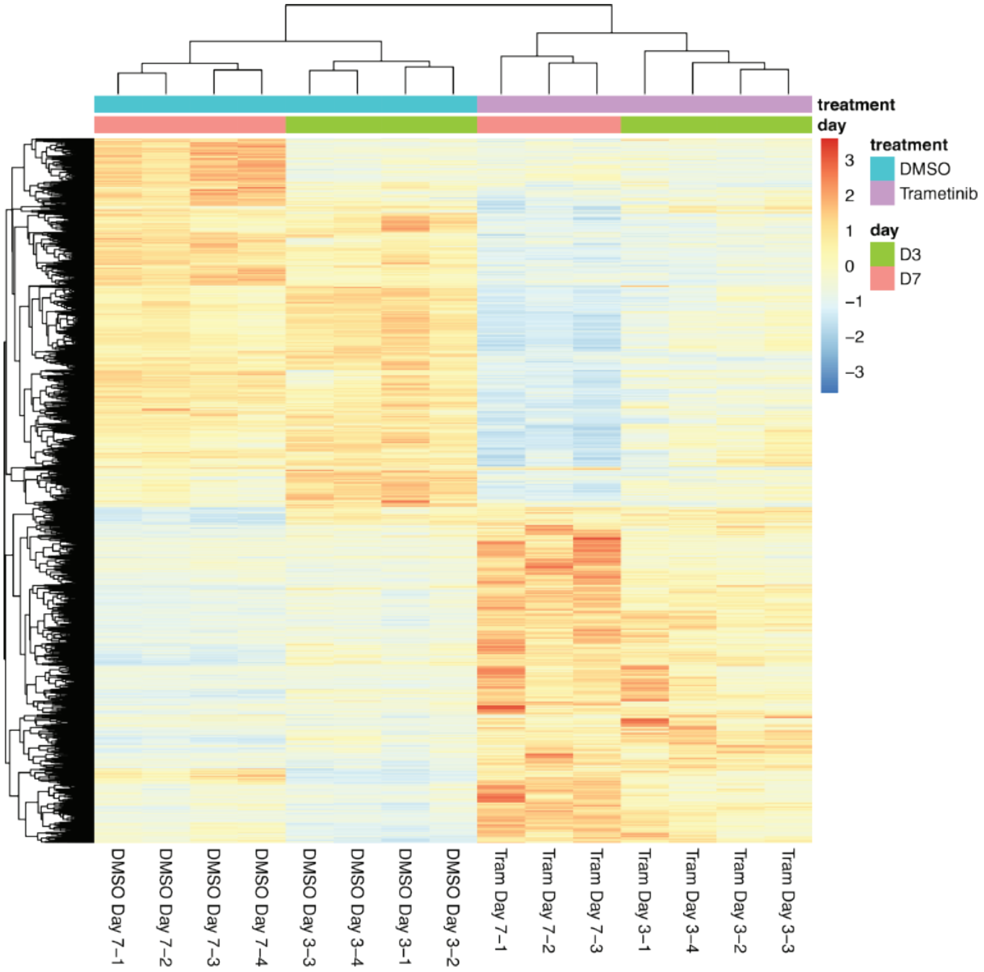

Borlase et al. Suplementary Figure 3

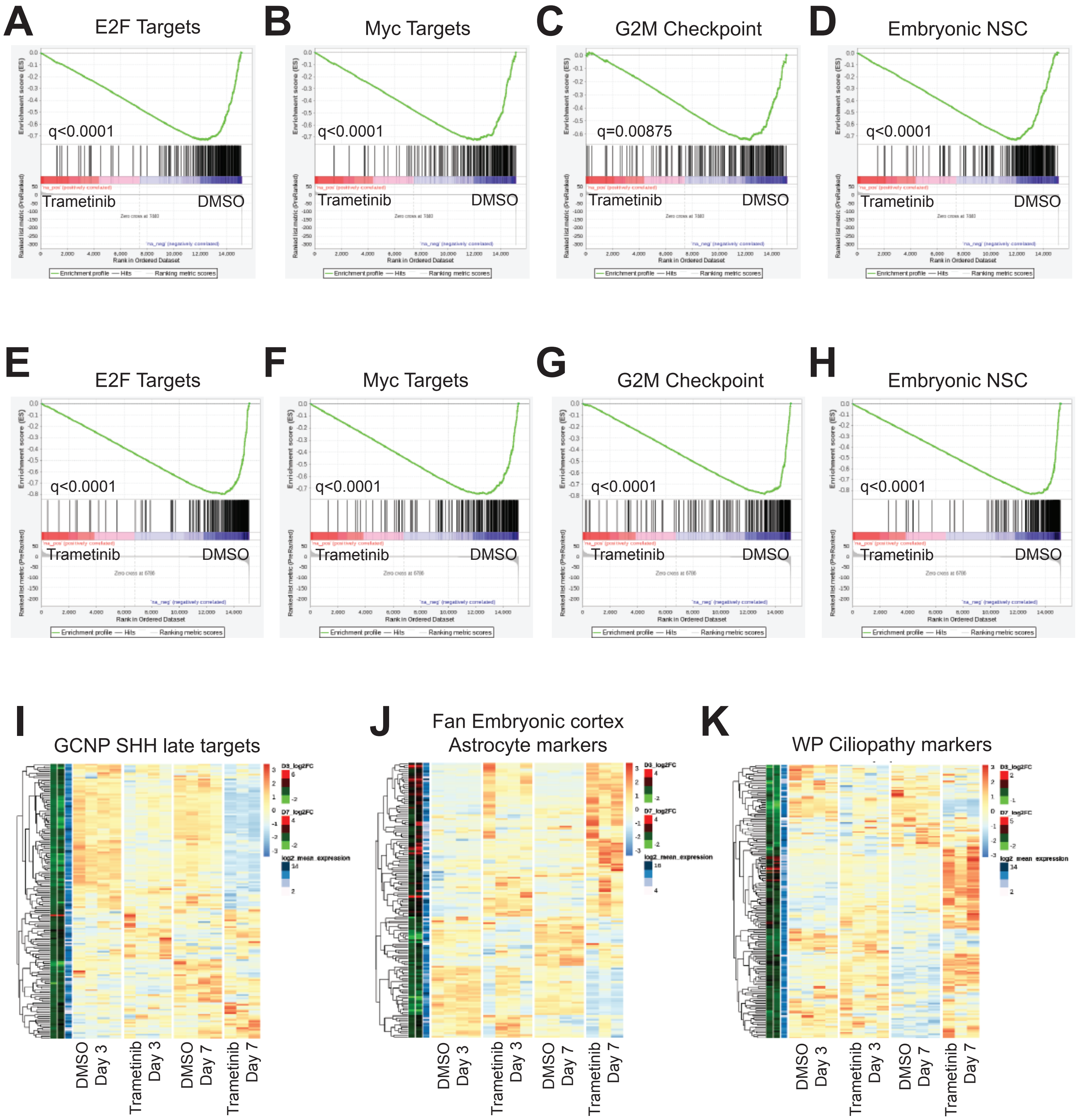

A

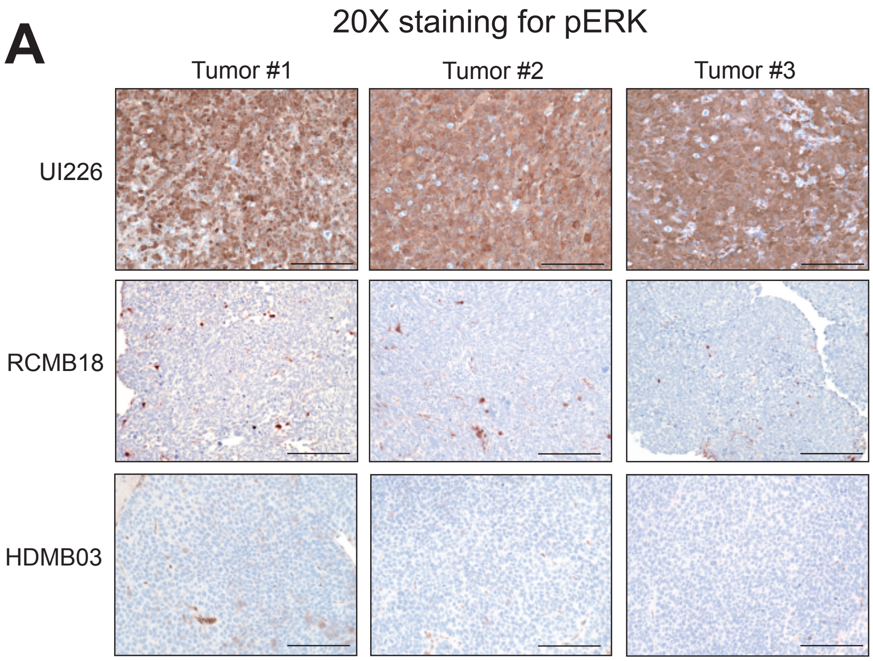
20X staining for pERK
Tumor #1
Tumor #2
Tumor #3
UI226
RCMB18
HDMB03

B

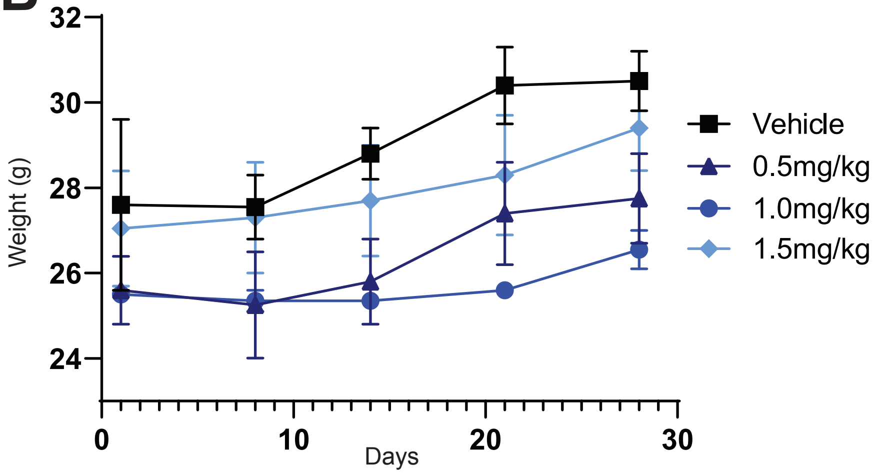
32
30
28
26
24
Weight (g)
0
10
20
30
Days
Vehicle
0.5mg/kg
1.0mg/kg
1.5mg/kg
