# Supplementary figures and images for: Cross-species analysis of SHH medulloblastoma models reveals significant inhibitory effects of trametinib on tumor progression
Source: Cell Death Discov. 2023 Sep 19;9:347. doi: 10.1038/s41420-023-01646-0 (PMC10509237; doi:10.1038/s41420-023-01646-0)

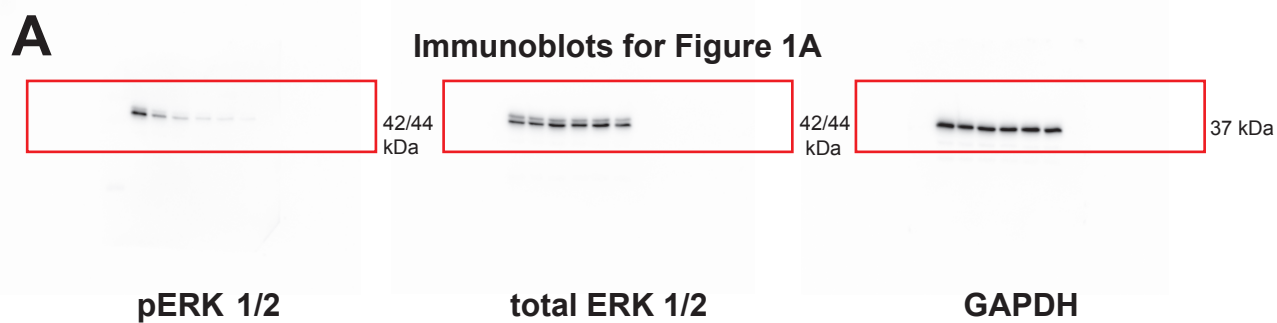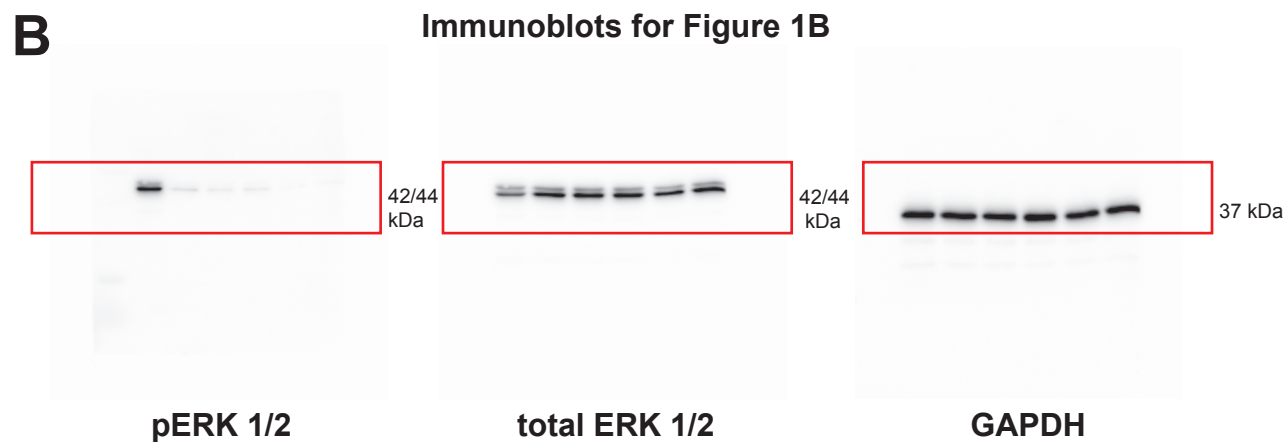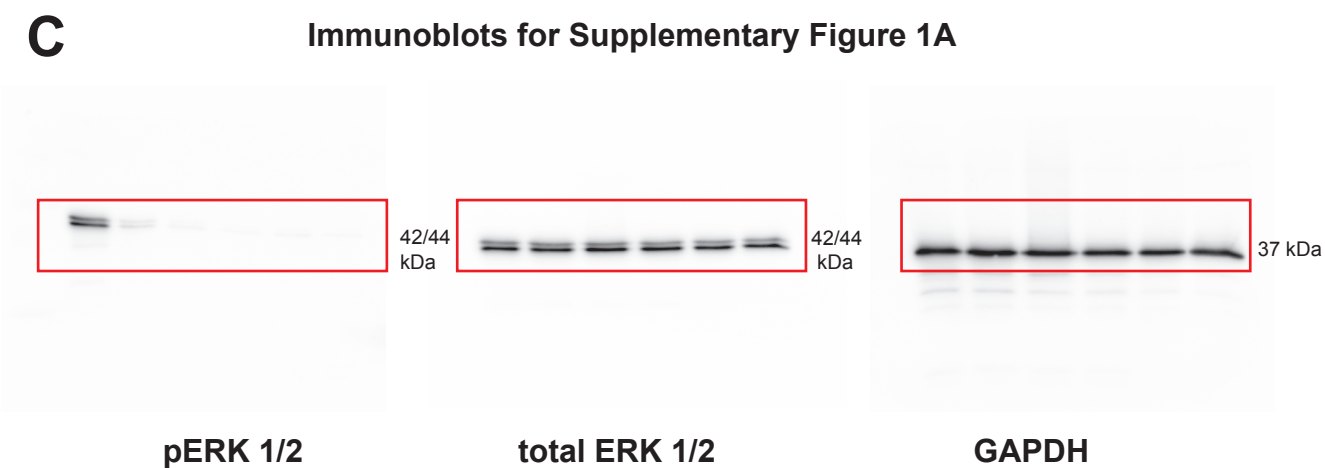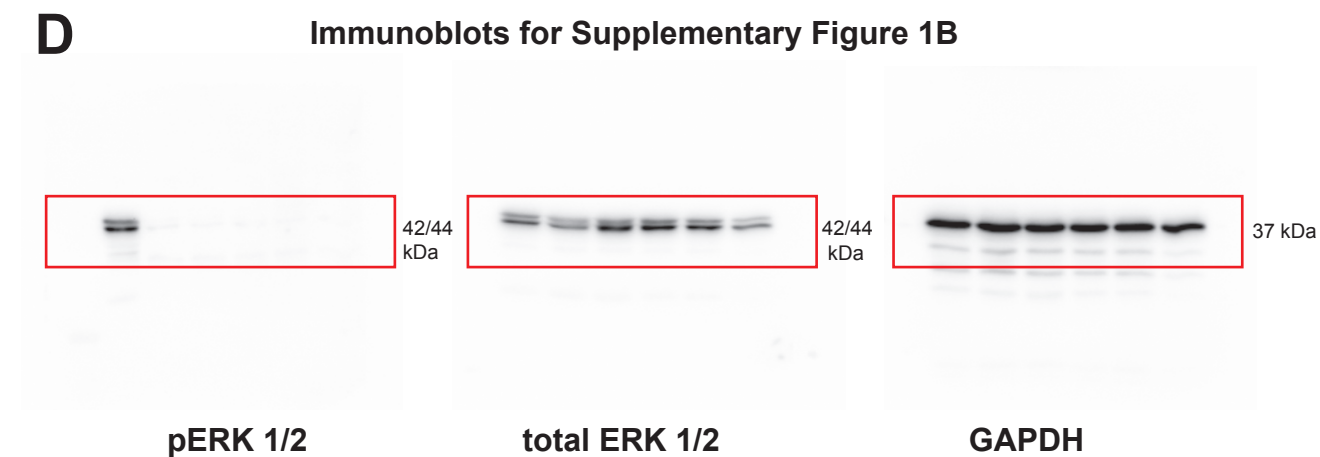

Supplement: Supplementary file 3 — Original Data File [file 41420_2023_1646_MOESM3_ESM.pdf]
