# Supplementary material for: Cross-species analysis of SHH medulloblastoma models reveals significant inhibitory effects of trametinib on tumor progression
Source: Cell Death Discov. 2023 Sep 19;9:347. doi: 10.1038/s41420-023-01646-0 (PMC10509237; doi:10.1038/s41420-023-01646-0)
Supplement: Supplementary file 5 — Supplementary Table - antibodies [file 41420_2023_1646_MOESM5_ESM.docx]

**Supplementary Table 1: List of antibodies used in this study**

| **Immunoblotting** | | |
| --- | --- | --- |
| Name | Company/Catalogue number | Dilution |
| p-ERK | CST (4370) | 1/1000 |
| ERK | CST (4695) | 1/1000 |
| GAPDH | Santa Cruz (sc47724) | 1/1000 |
| **Immunoblotting secondary antibodies** | | |
| Goat anti-mouse HRP | Abcam (ab6789) | 1/3000 |
| Donkey anti-rabbit HRP | Jackson ImmunoResearch (711-035-152) | 1/5000 |
| **Immunohistochemistry** | | |
| Anti-mitochondria | Abcam (ab92824) | 1/500 |
| p-ERK | CST (9101) | 1/400 |
| **Immunohistochemistry secondary antibodies** | | |
| Biotin-SP goat anti-rabbit | Cedarlane (111-065-144) | 1/500 |
| Biotin-SP sheep anti-mouse | Jackson ImmunoResearch (515-065-003) | 1/500 |
